# Supplementary material for: Pyrazole-Enriched Cationic Nanoparticles Induced Early- and Late-Stage Apoptosis in Neuroblastoma Cells at Sub-Micromolar Concentrations
Source: Pharmaceuticals (Basel). 2023 Mar 5;16(3):393. doi: 10.3390/ph16030393 (PMC10056113; doi:10.3390/ph16030393)
Supplement: Supplementary file 1 [file pharmaceuticals-16-00393-s001.zip › pharmaceuticals-2254586-SM-R1.pdf]

pharmaceuticals

Supplementary Materials

Pyrazole-Enriched Cationic Nanoparticles Induced Early- and Late-Stage Apoptosis in Neuroblastoma Cells at Sub-Micromolar Concentrations

Guendalina Zuccari [1], Alessia Zorzoli [2], Danilo Marimpietri [2], Chiara Brullo [3] and Silvana Alfei [1,] *

[1] Department of Pharmacy, Section of Chemistry and Pharmaceutical and Food Technologies, University of Genoa, Viale Cembrano, 4, 16148 Genoa, Italy; zuccari@difar.unige.it (G.Z.)
[2] Cell Factory, IRCCS Istituto Giannina Gaslini, via Gerolamo Gaslini 5, 16147 Genoa, Italy; alessiazorzoli@gaslini.org (A.Z.), danilomarimpietri@gaslini.org (D.M.)
[3] Department of Pharmacy (DIFAR), Section of Medicinal Chemistry and Cosmetic Product, University of Genoa, Viale Benedetto XV, 3, 16132 Genoa, chiara.brullo@unige.it (C.B.)
* Correspondence: alfei@difar.unige.it (S.A.); Tel.: +39-010-355-2296 (S.A.)

Section S1. Structure of compounds reported previously.

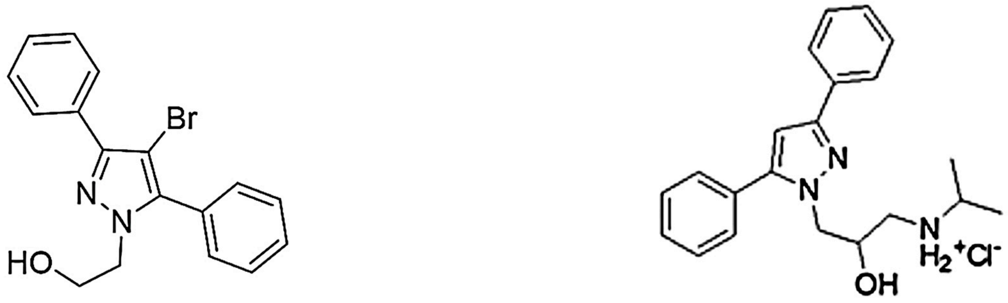

BBB4 CB1H

(**a**) (**b**)

Figure S1. Structure of BBB4 (**a**); structure of CB1H (**b**).

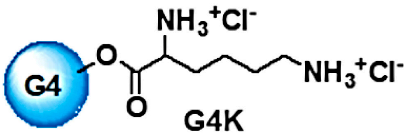

(**a**)

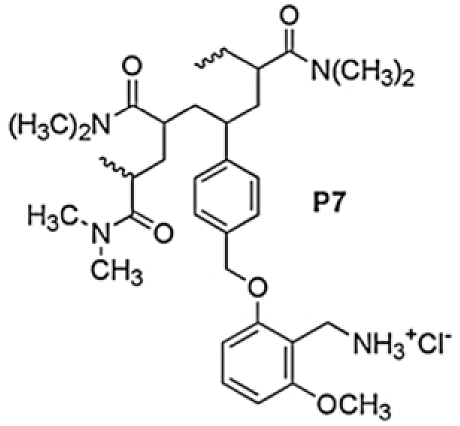

(**b**)

Figure S2. Structure of cationic dendrimer G4K (**a**); structure of cationic copolymer P7 (**b**).

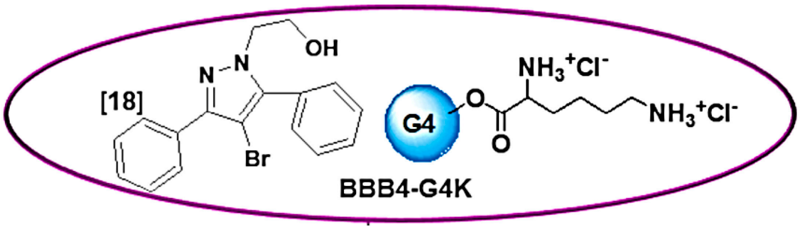

(**a**)

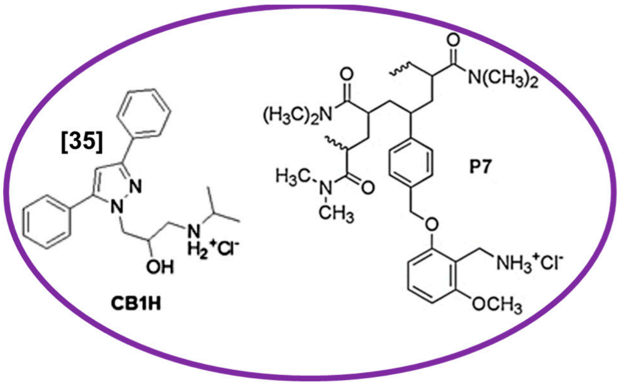

(**b**)

Figure S3. Intuitive representations of the structure of BBB4 G4K NPs (**a**) and of the structure of CB1H P7 NPs (**b**). Numbers in square brackets show the number of moles of pyrazoles which have been loaded per mole of dendrimer G4K (**a**) or of copolymer P7 (**b**).

Section S2. Cytotoxicity Studies.

Table S1. Concentrations of each sample administered to NB cells.

Samples	Concentrations (μM)									
BBB4	0	0.5	1	2.5	5	7.5	10	12.5	15	20
BBB4-G4K	0	0.02810	0.0563	0.1407	0.2814	0.4221	0.5628	0.7034	0.8441	1.1255
G4K	0	0.03280	0.0656	0.1640	0.3281	0.4921	0.6562	0.8202	0.8484	1.3123
CB1H	0	1	5	10	15	20	25	50	75	100
CB1H-P7	0	0.02874	0.1437	0.2874	0.4311	0.5748	0.7185	1.4370	2.1555	2.8740
P7	0	0.02867	0.1433	0.2867	0.4300	0.5734	0.7167	1.4334	2.1501	2.8668

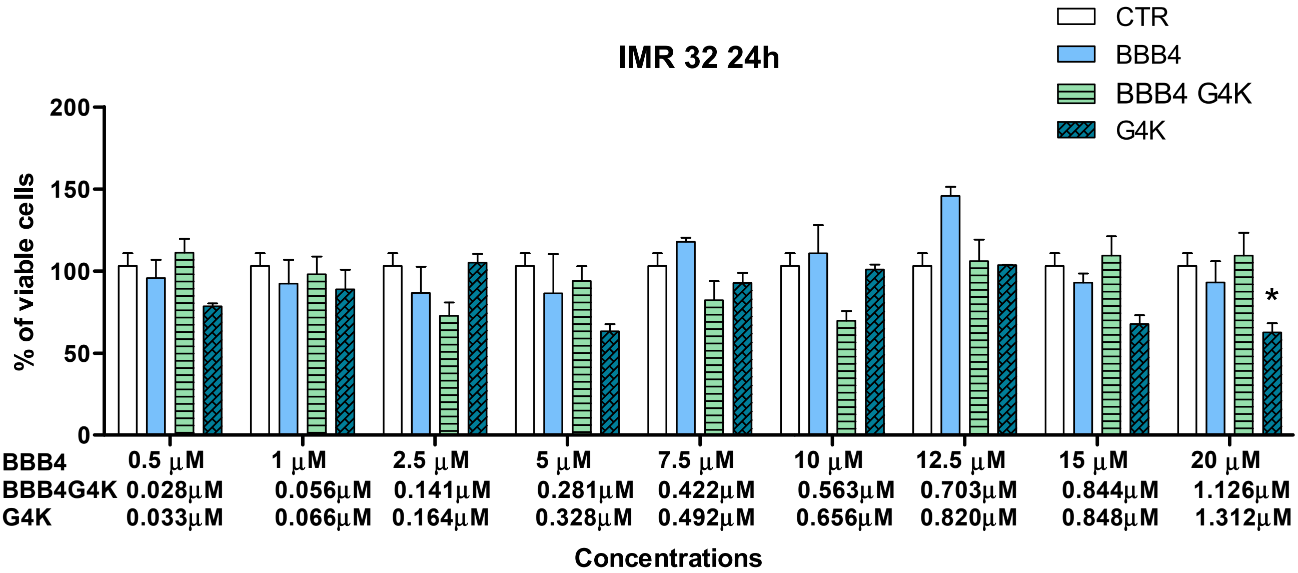
IMR 32 24h
CTR
BBB4
BBB4 G4K
G4K
% of viable cells
200
150
100
50
0
*
BBB4 0.5 μM 1 μM 2.5 μM 5 μM 7.5 μM 10 μM 12.5 μM 15 μM 20 μM
BBB4G4K 0.028μM 0.056μM 0.141μM 0.281μM 0.422μM 0.563μM 0.703μM 0.844μM 1.126μM
G4K 0.033μM 0.066μM 0.164μM 0.328μM 0.492μM 0.656μM 0.820μM 0.848μM 1.312μM
Concentrations

(a)

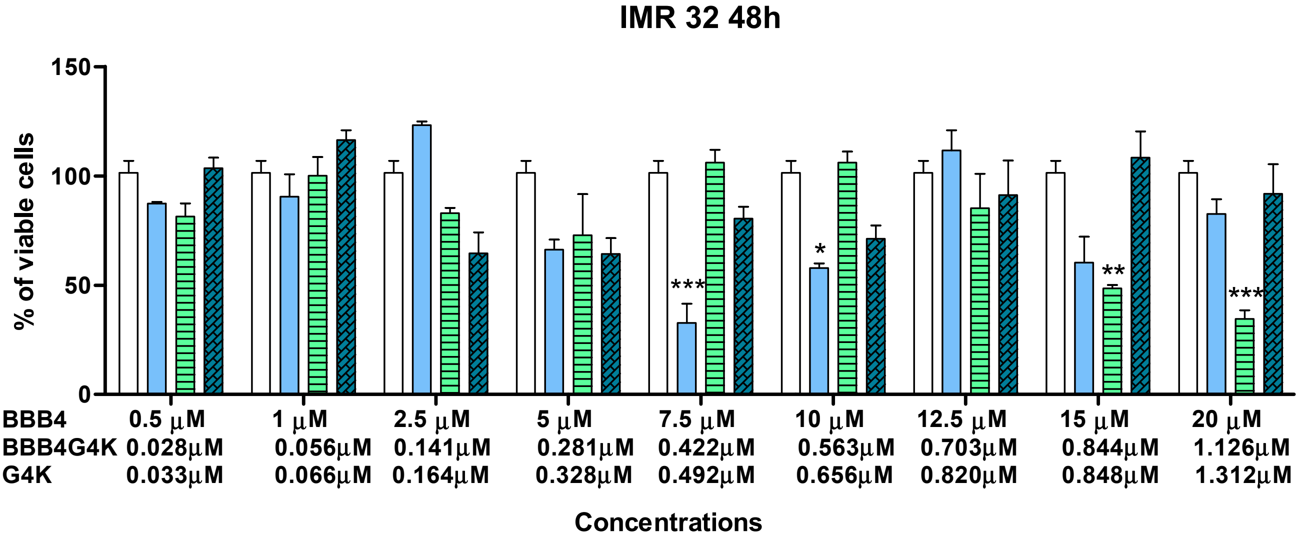
IMR 32 48h
% of viable cells
150
100
50
0
***
*
**
***
BBB4 0.5 μM 1 μM 2.5 μM 5 μM 7.5 μM 10 μM 12.5 μM 15 μM 20 μM
BBB4G4K 0.028μM 0.056μM 0.141μM 0.281μM 0.422μM 0.563μM 0.703μM 0.844μM 1.126μM
G4K 0.033μM 0.066μM 0.164μM 0.328μM 0.492μM 0.656μM 0.820μM 0.848μM 1.312μM
Concentrations

(b)

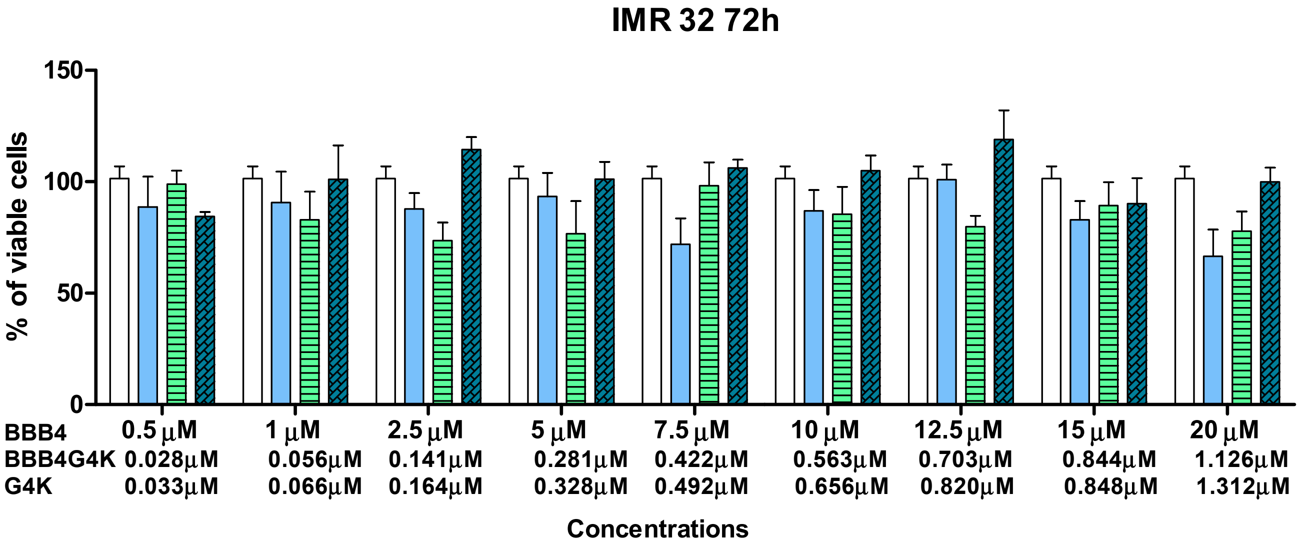

(**c**)

Figure S4. Dose- and time-dependent cytotoxicity activity of G4K, BBB4, and BBB4-G4K NPs at 24 h (**a**), 48 h (**b**), and 72 h (**c**) towards IMR 32 cells. Significance refers to control (CTR) ($p > 0.05$ ns; $p < 0.05$ *; $p < 0.01$ **; $p < 0.001$ ***).

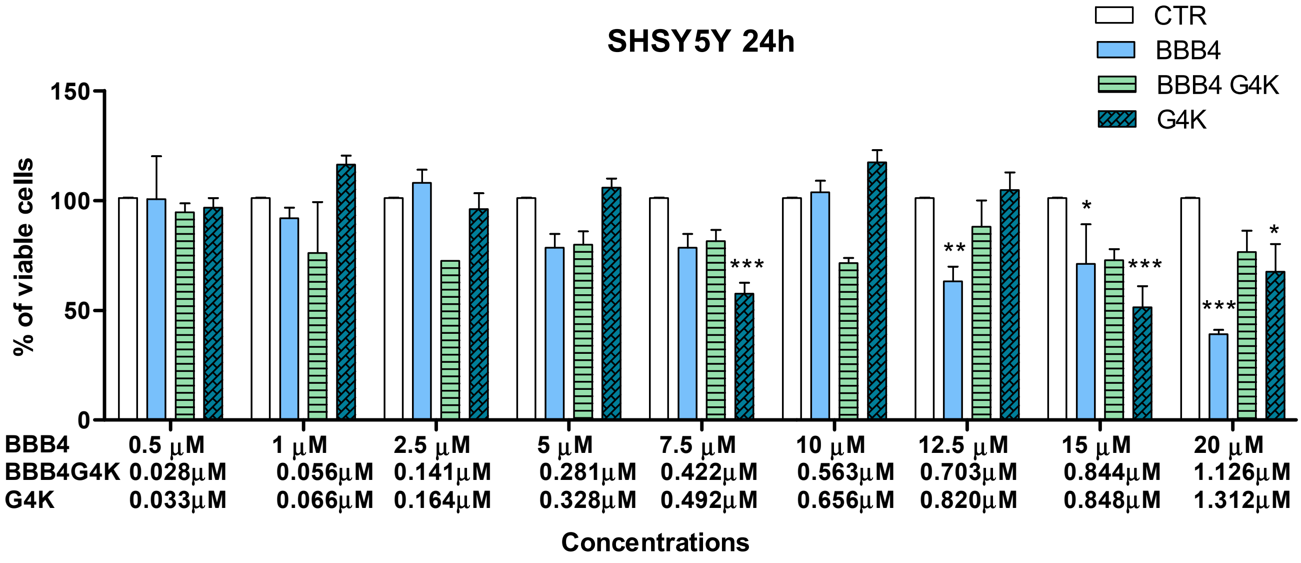

(**a**)

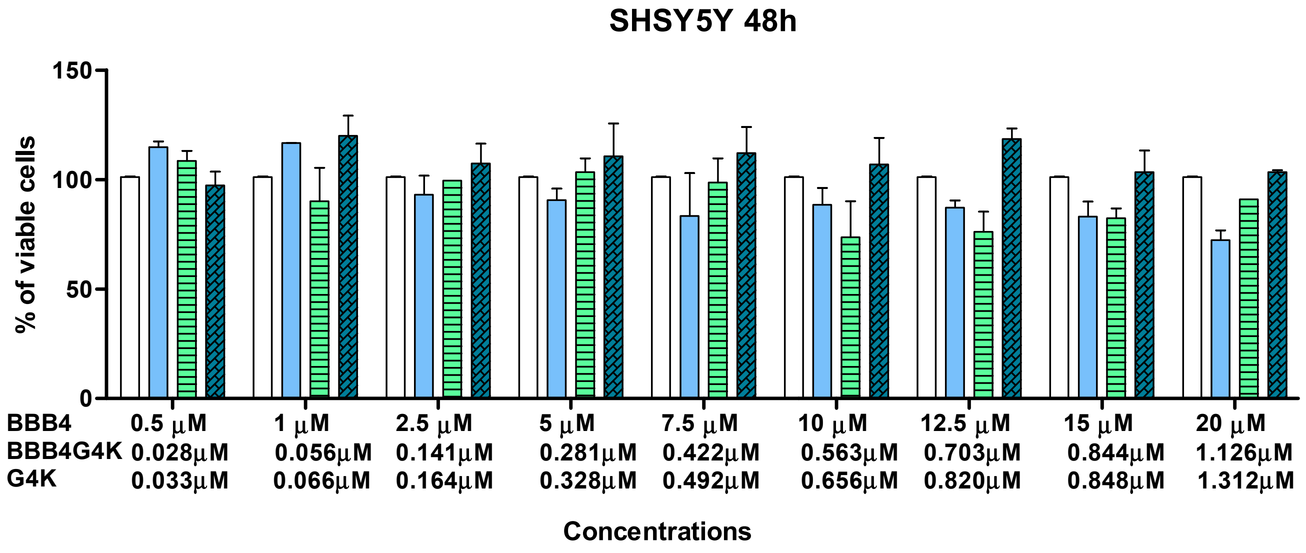

(**b**)

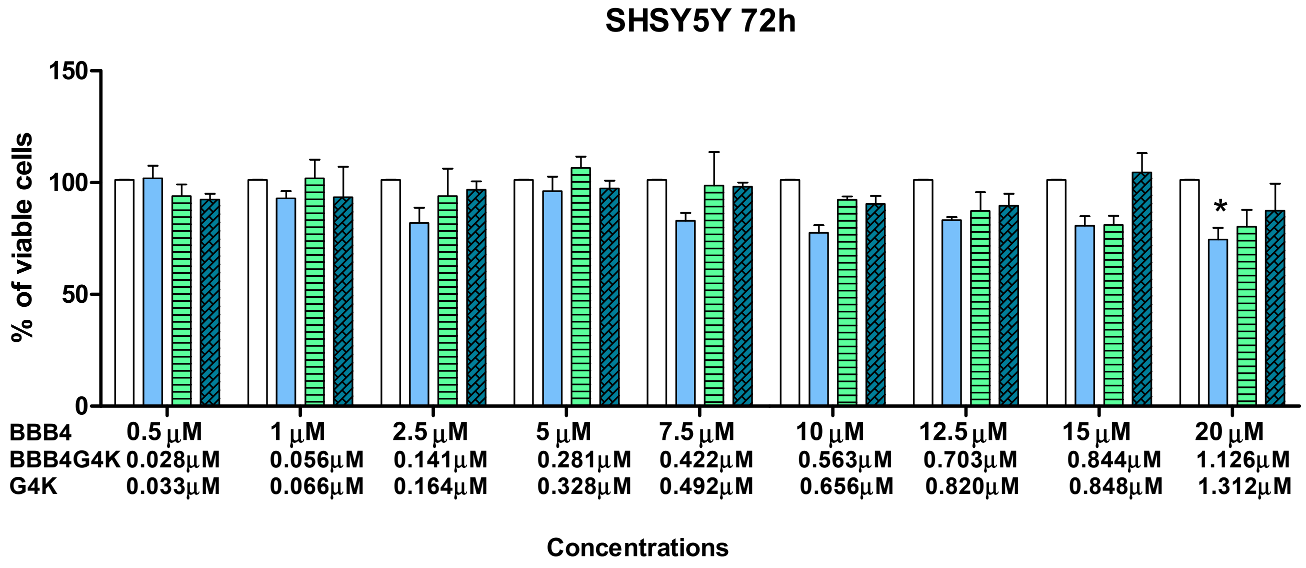

(**c**)

Figure S5. Dose- and time-dependent cytotoxicity activity of G4K, BBB4, and BBB4-G4K NPs at 24 h (**a**), 48 h (**b**), and 72 h (**c**) towards SHSY 5Y cells. Significance refers to control ($p > 0.05$ ns; $p < 0.05$ *; $p < 0.01$ **; $p < 0.001$ ***).

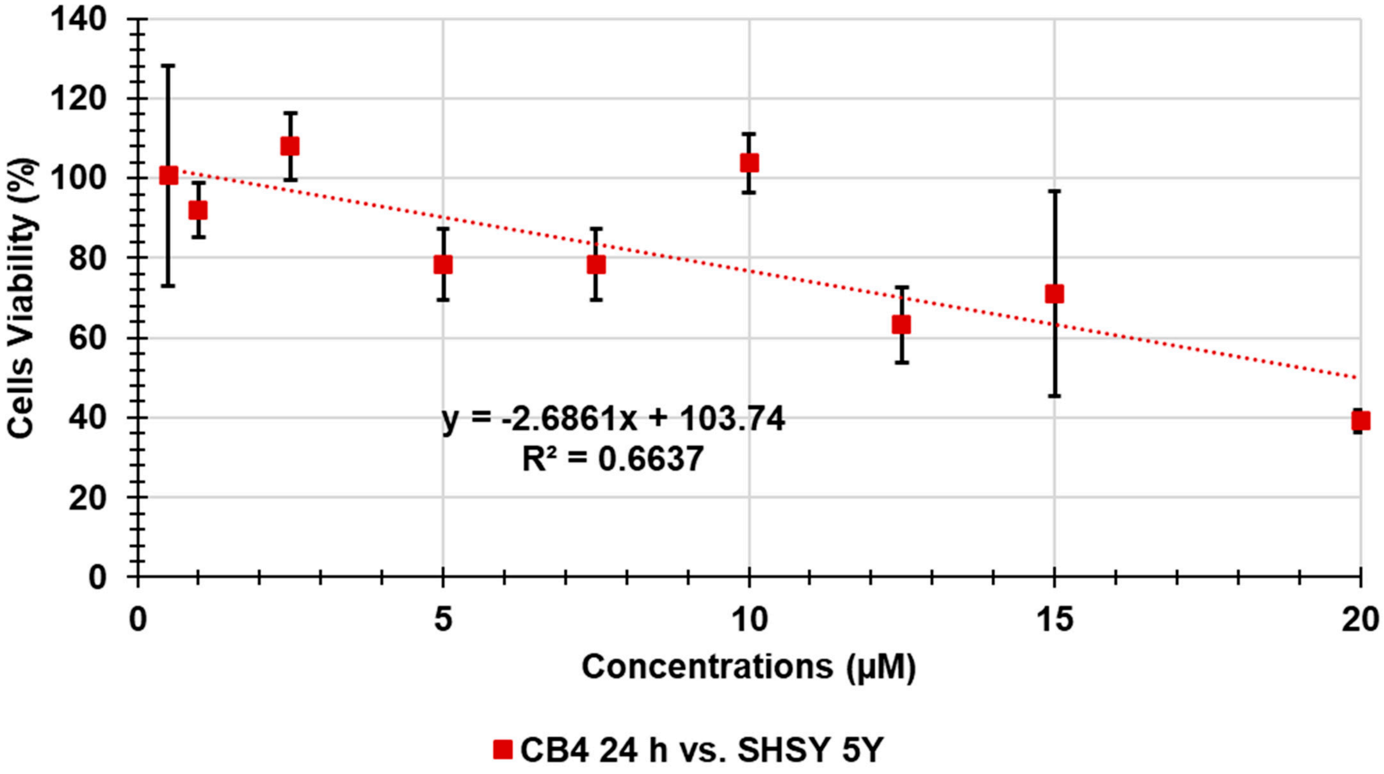

Figure S6. Linear regression model fitting the dispersion graph obtained reporting in graph the viability % of SHSY 5Y cells vs. the concentrations of BBB4 at 24 h of exposure.

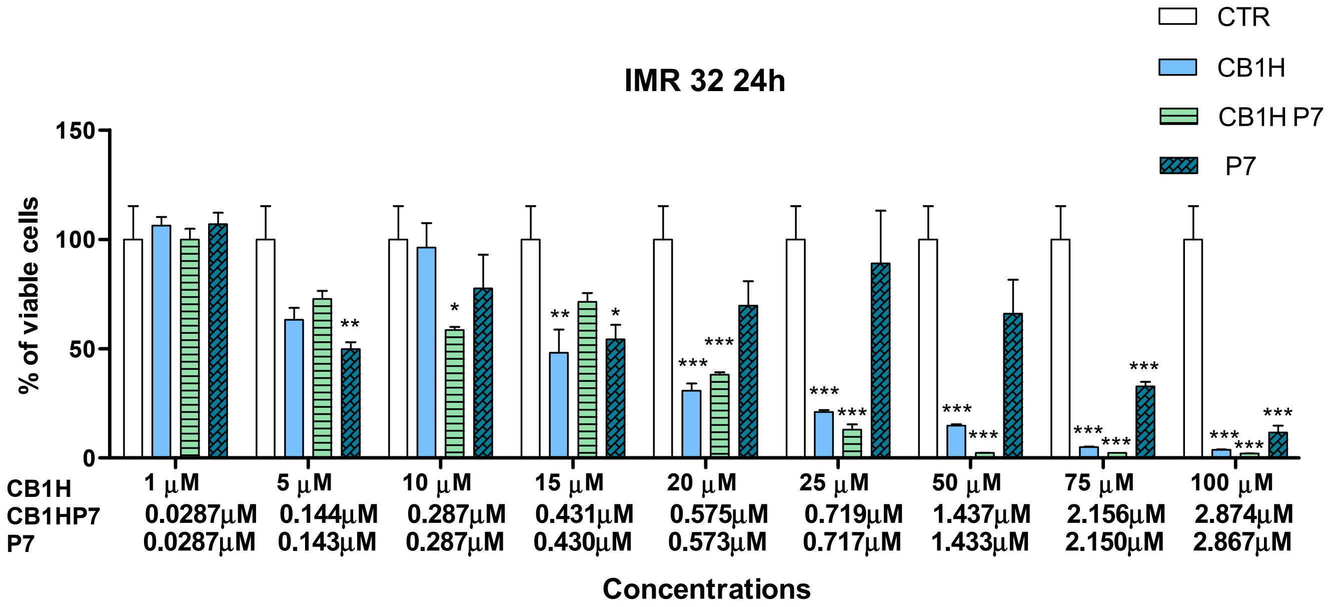
CTR
CB1H
CB1H P7
P7
IMR 32 24h
150
100
50
0
% of viable cells
CB1H
CB1HP7
P7
1 μM
5 μM
10 μM
15 μM
20 μM
25 μM
50 μM
75 μM
100 μM
0.0287μM 0.144μM 0.287μM 0.431μM 0.575μM 0.719μM 1.437μM 2.156μM 2.874μM
0.0287μM 0.143μM 0.287μM 0.430μM 0.573μM 0.717μM 1.433μM 2.150μM 2.867μM
Concentrations

(**a**)

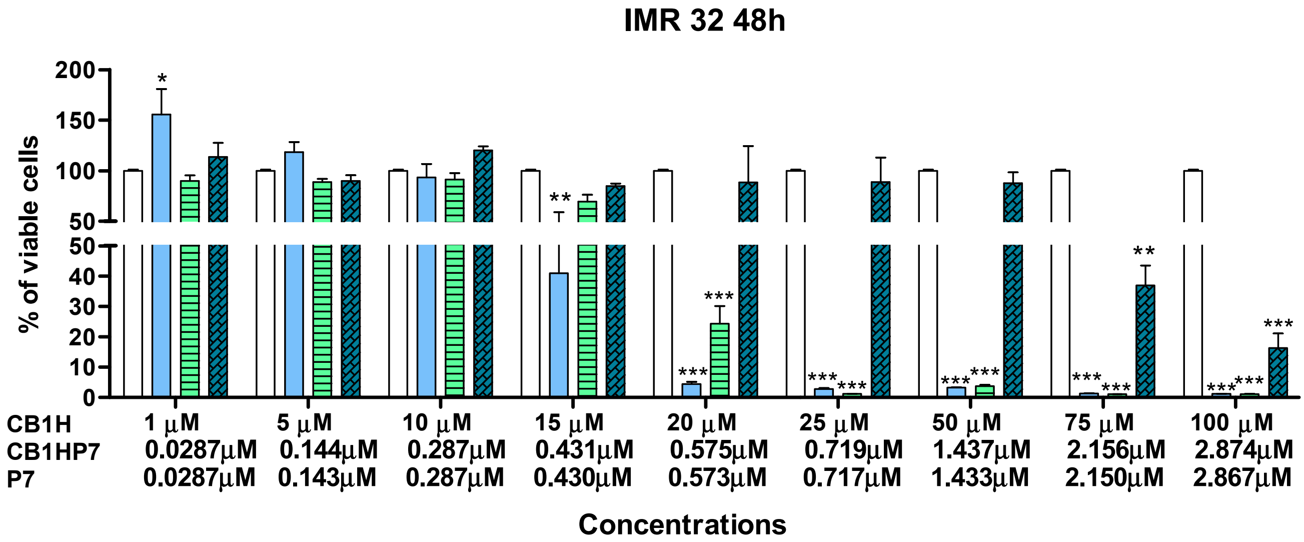
IMR 32 48h
200
150
100
50
40
30
20
10
0
% of viable cells
CB1H
CB1HP7
P7
1 μM
5 μM
10 μM
15 μM
20 μM
25 μM
50 μM
75 μM
100 μM
0.0287μM 0.144μM 0.287μM 0.431μM 0.575μM 0.719μM 1.437μM 2.156μM 2.874μM
0.0287μM 0.143μM 0.287μM 0.430μM 0.573μM 0.717μM 1.433μM 2.150μM 2.867μM
Concentrations

(**b**)

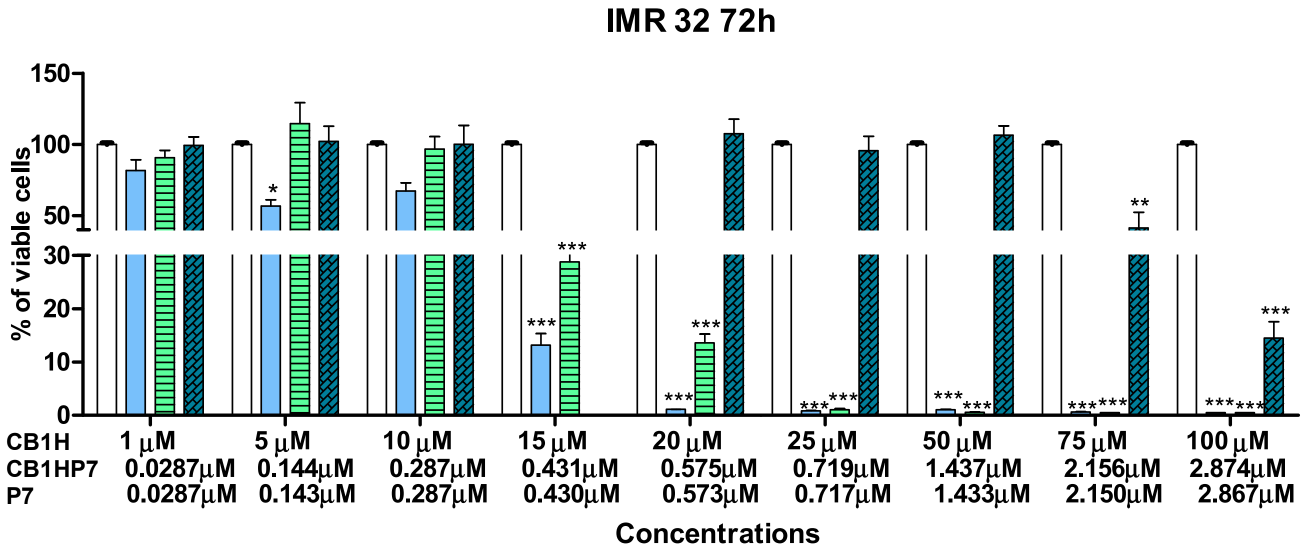

(**c**)

Figure S7. Dose- and time-dependent cytotoxicity activity of CB1H, P7 and CB1H-P7 NPs, at 24 h (**a**), 48 h (**b**), and 72 h (**c**) towards IMR-32 cells. Significance refers to control (p > 0.05 ns; p < 0.05 *; p < 0.01 **; p < 0.001 ***).

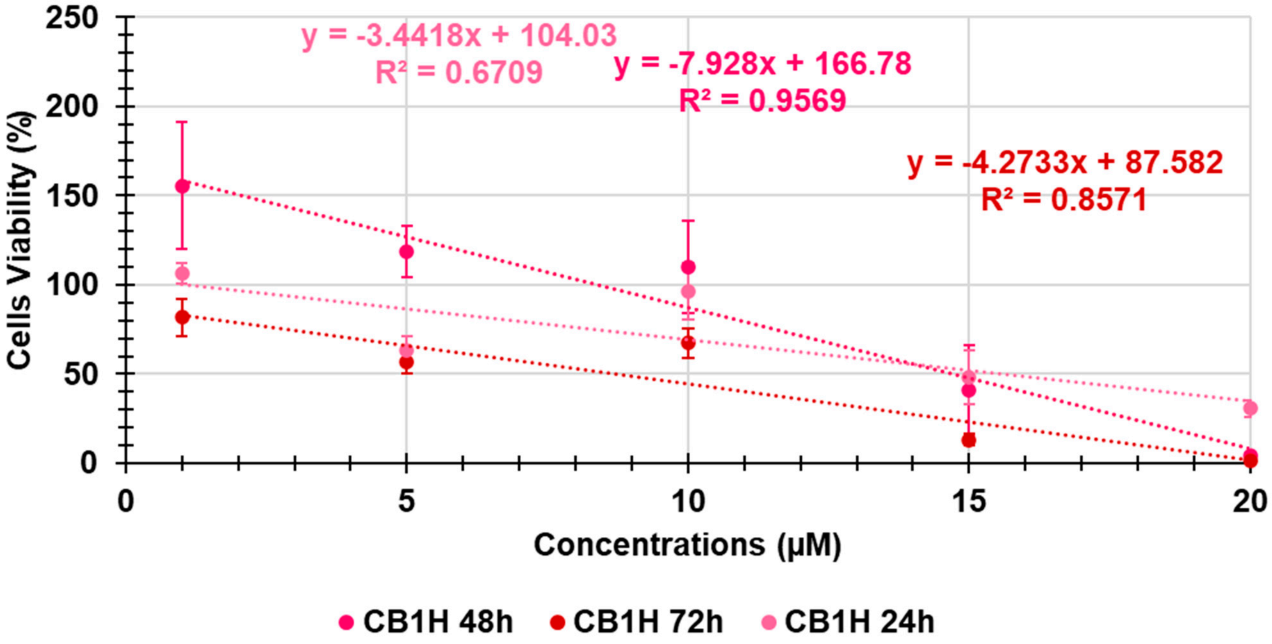

Figure S8. Dose-dependent cytotoxicity activity of CB1H at 24 h (pink), 48 h (dark pink), and 72 h (red) towards IMR-32 cells in the range 1-20 µM.

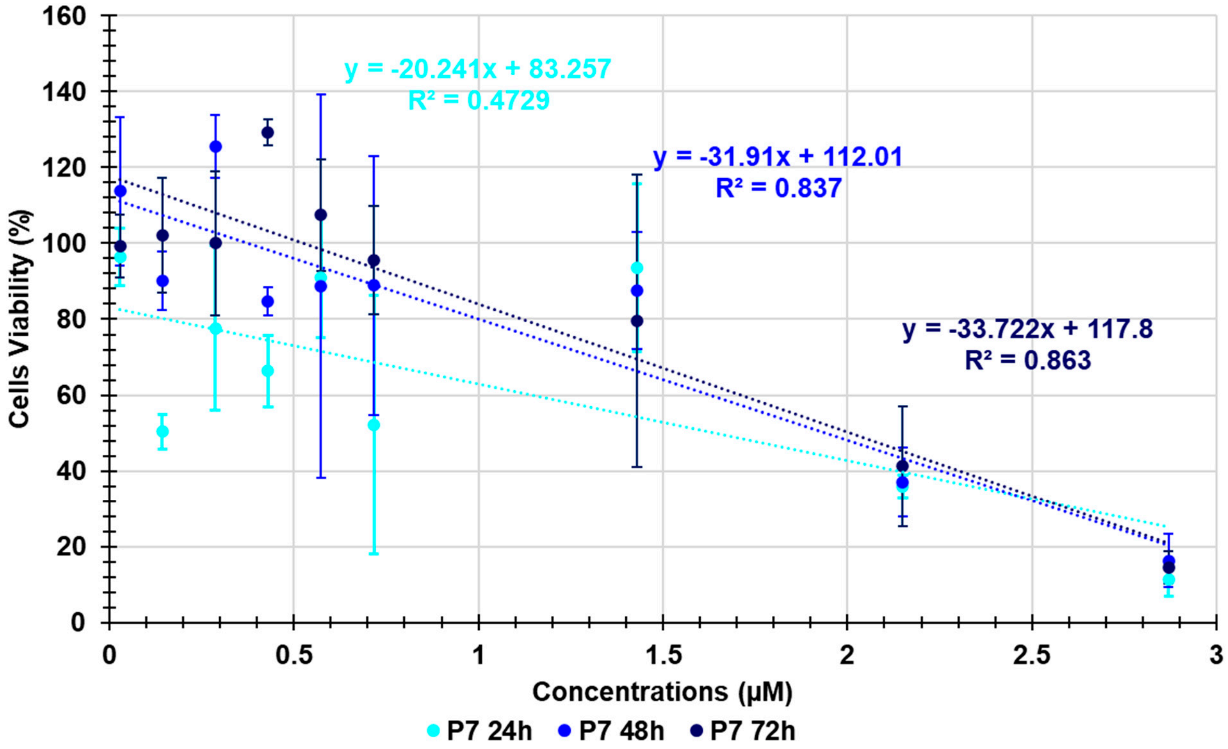

Figure S9. Dose-dependent cytotoxicity activity of P7 at 24 h (light blue), 48 h (blue), and 72 h (dark blue) towards IMR-32 cells in the range 1-2.8 µM.

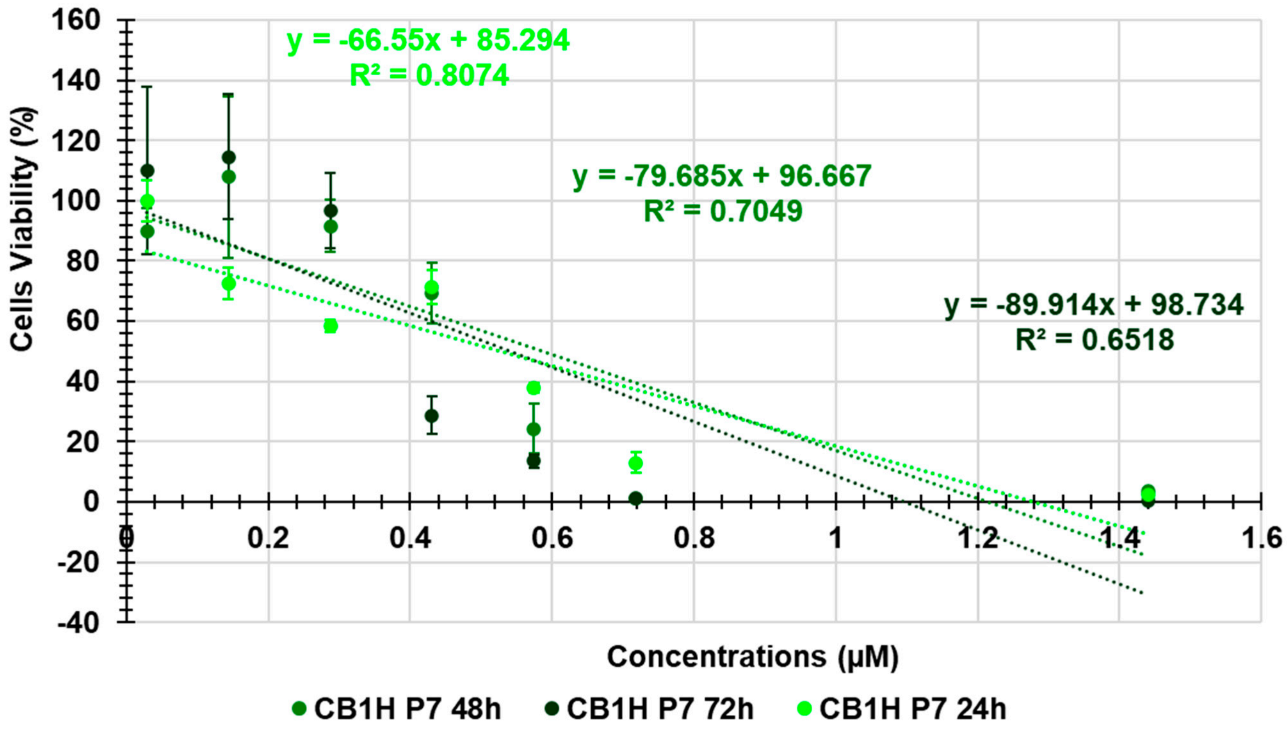

Figure S10. Dose-dependent cytotoxicity activity of CB1H-P7 NPs at 24 h (light green), 48 h (green), and 72 h (dark green) towards IMR-32 cells in the range 1-1.44 µM.

Table S2. IC_{50} of CB1H, P7 and CB1H-P7 NPs towards IMR-32 NB cells and normal human keratinocytes (HaCaT) at 24-, 48- and 72-hours exposition and their SI values.

Times (h)	CB1H (µM)	P7 NPs (µM)	CB1H-P7 NPs (µM)
24	15.70	1.64	0.53
48	14.73	1.94	0.59
72	8.79	2.01	0.54
	IC_{50} HaCaT (µM)		
24	57.30	2.10	1.50
48	42.69	2.38	1.42
72	47.63	1.71	1.33
	Selectivity Index		
24	3.6	1.3	2.8
48	2.9	1.2	2.4
72	5.4	0.9	2.5

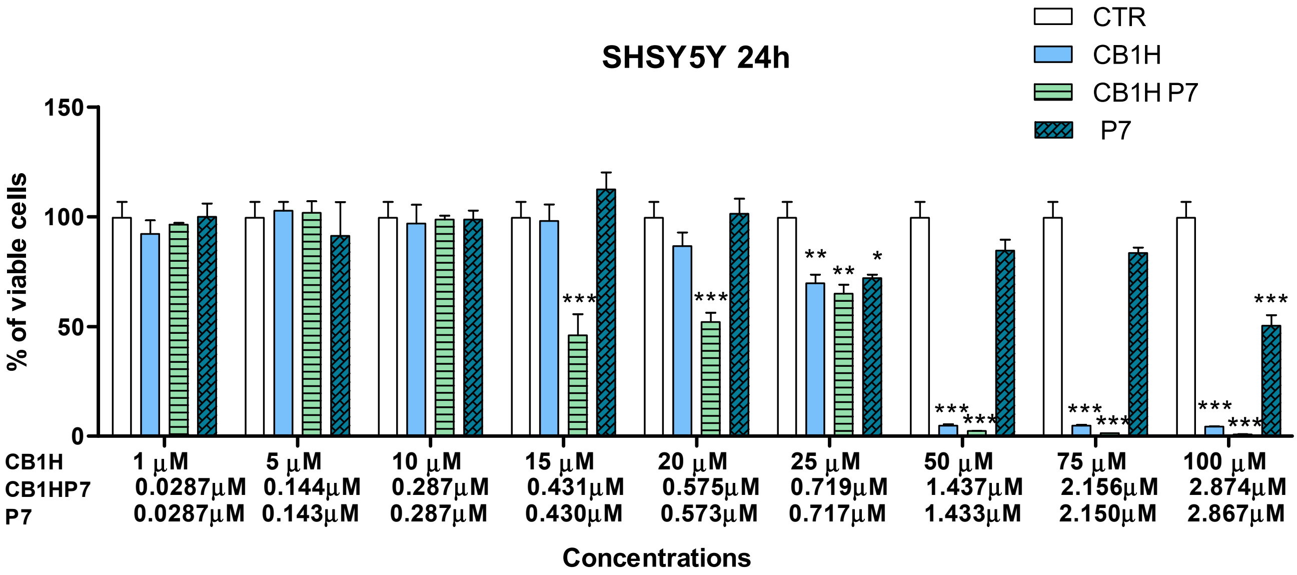
CTR
CB1H
CB1H P7
P7
SHSY5Y 24h
% of viable cells
150
100
50
0
CB1H
CB1HP7
P7
1 µM 0.0287µM 0.0287µM
5 µM 0.144µM 0.143µM
10 µM 0.287µM 0.287µM
15 µM 0.431µM 0.430µM
20 µM 0.575µM 0.573µM
25 µM 0.719µM 0.717µM
50 µM 1.437µM 1.433µM
75 µM 2.156µM 2.150µM
100 µM 2.874µM 2.867µM
Concentrations

(a)

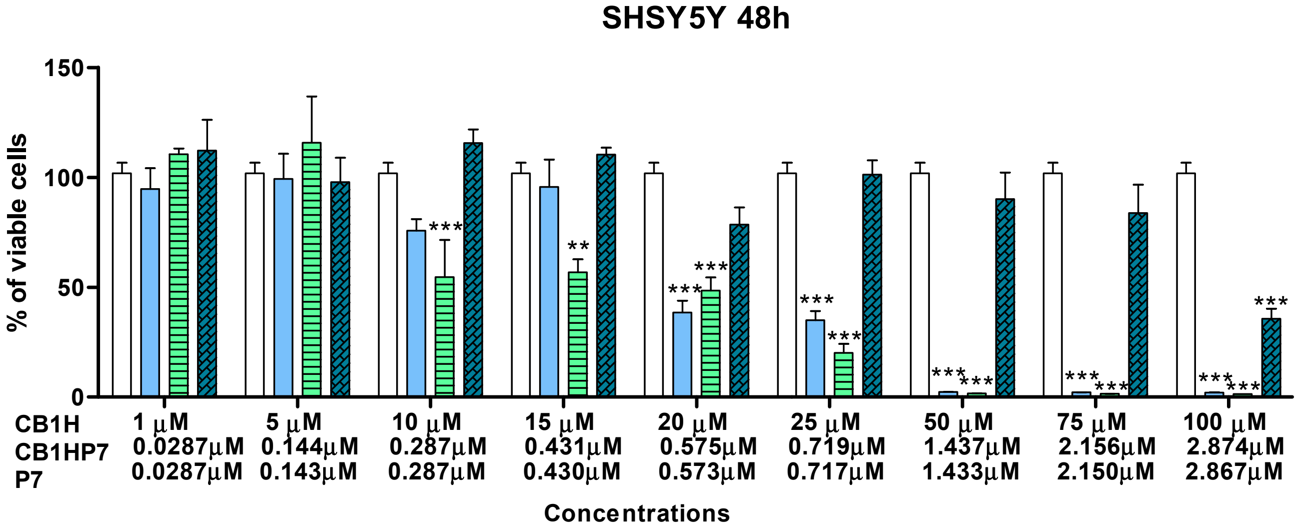
SHSY5Y 48h
% of viable cells
150
100
50
0
CB1H
CB1HP7
P7
1 µM 0.0287µM 0.0287µM
5 µM 0.144µM 0.143µM
10 µM 0.287µM 0.287µM
15 µM 0.431µM 0.430µM
20 µM 0.575µM 0.573µM
25 µM 0.719µM 0.717µM
50 µM 1.437µM 1.433µM
75 µM 2.156µM 2.150µM
100 µM 2.874µM 2.867µM
Concentrations

(b)

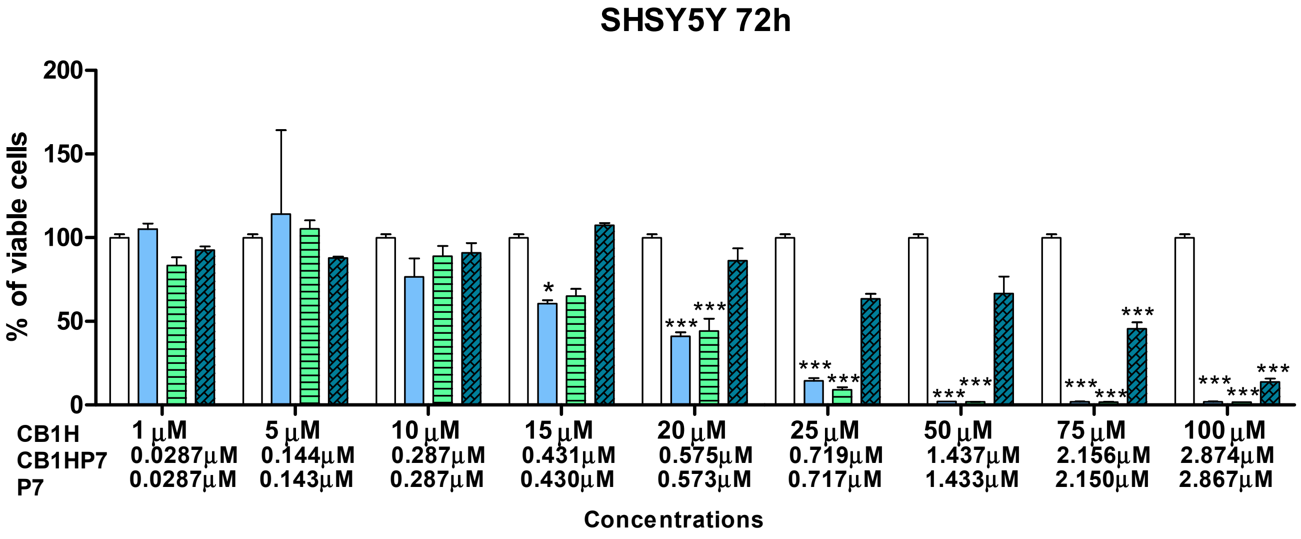

(**c**)

Figure S11. Dose- and time-dependent cytotoxicity activity of CB1H, P7, and CB1H-P7 at 24 h (**a**), 48 h (**b**), and 72 h (**c**) towards SHSY 5Y cells. Significance refers to control (p > 0.05 ns; p < 0.05 *; p < 0.01 **; p < 0.001 ***).

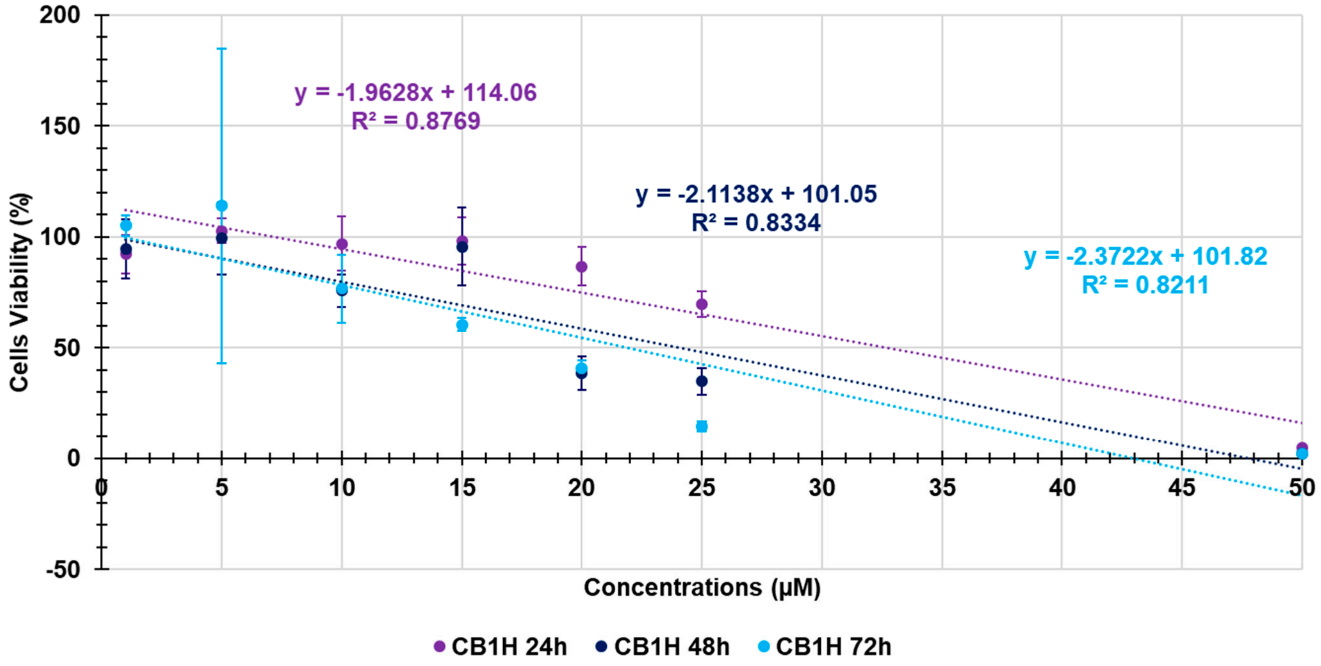

Figure S12. Dose-dependent cytotoxicity activity of CB1H at 24 h (purple), 48 h (blue), and 72 h (light blue) towards SHSY 5Y cells in the range 1-50 μM.

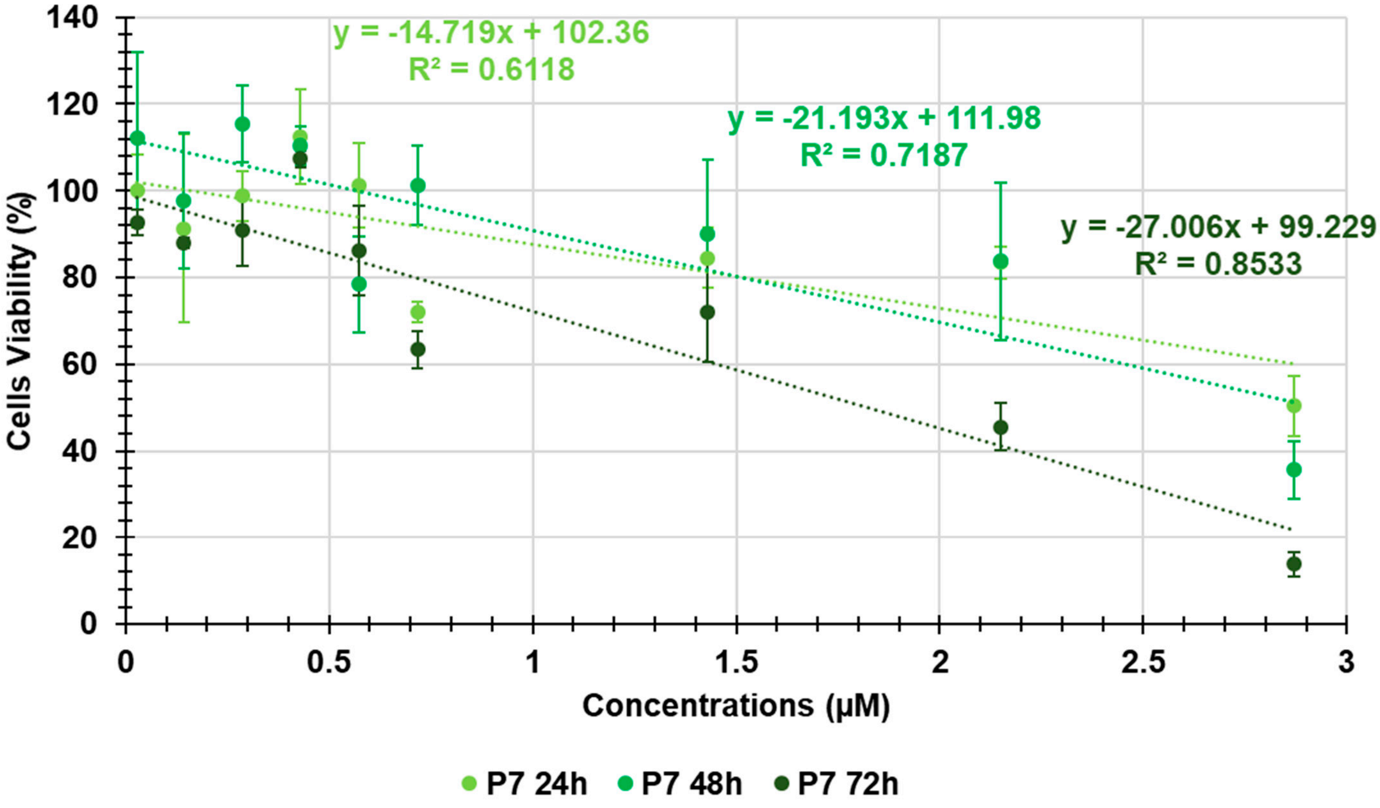

Figure S13. Dose-dependent cytotoxicity activity of P7 at 24 h (light green), 48 h (green), and 72 h (dark green) towards SHSY 5Y cells in the range 1-2.8 μM.

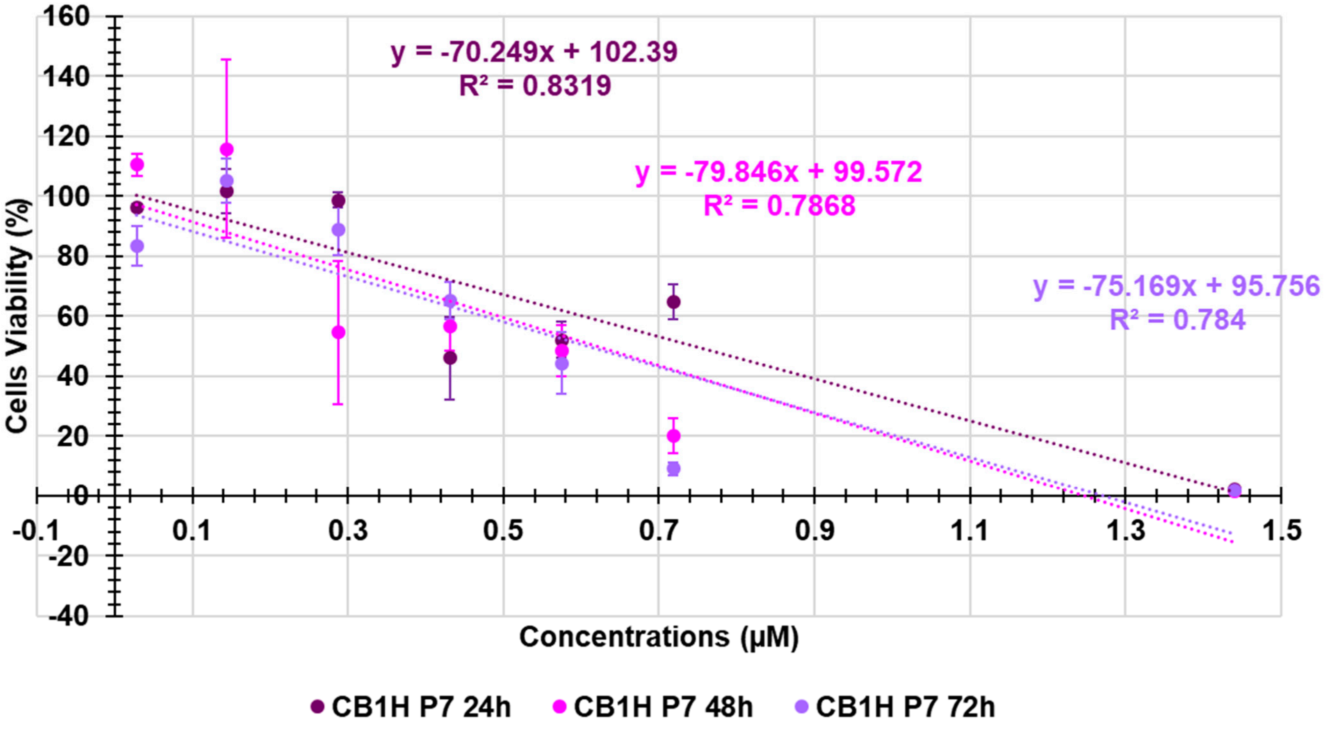

Figure S14. Dose-dependent cytotoxicity activity of CB1H-P7 NPs at 24 h (dark purple), 48 h (fuchsia), and 72 h (purple) towards SHSY 5Y cells in the range 1-1.44 μM.

Table S3. IC_{50} of CB1H, P7 and CB1H-P7 NPs towards SHSY 5Y NB cells and normal human keratinocytes (HaCaT) at 24-, 48- and 72-hours exposition and their SI values.

Times (h)	CB1H (μM)	P7 NPs (μM)	CB1H-P7 NPs (μM)
24	32.64	3.56	0.75
48	24.15	2.92	0.62
72	21.84	1.82	0.61
	IC_{50} HaCaT (μM)		
24	57.30	2.10	1.50
48	42.69	2.38	1.42
72	47.63	1.71	1.33
	Selectivity Index		
24	1.8	0.6	2.0
48	1.8	0.8	2.3
72	2.2	0.9	2.2

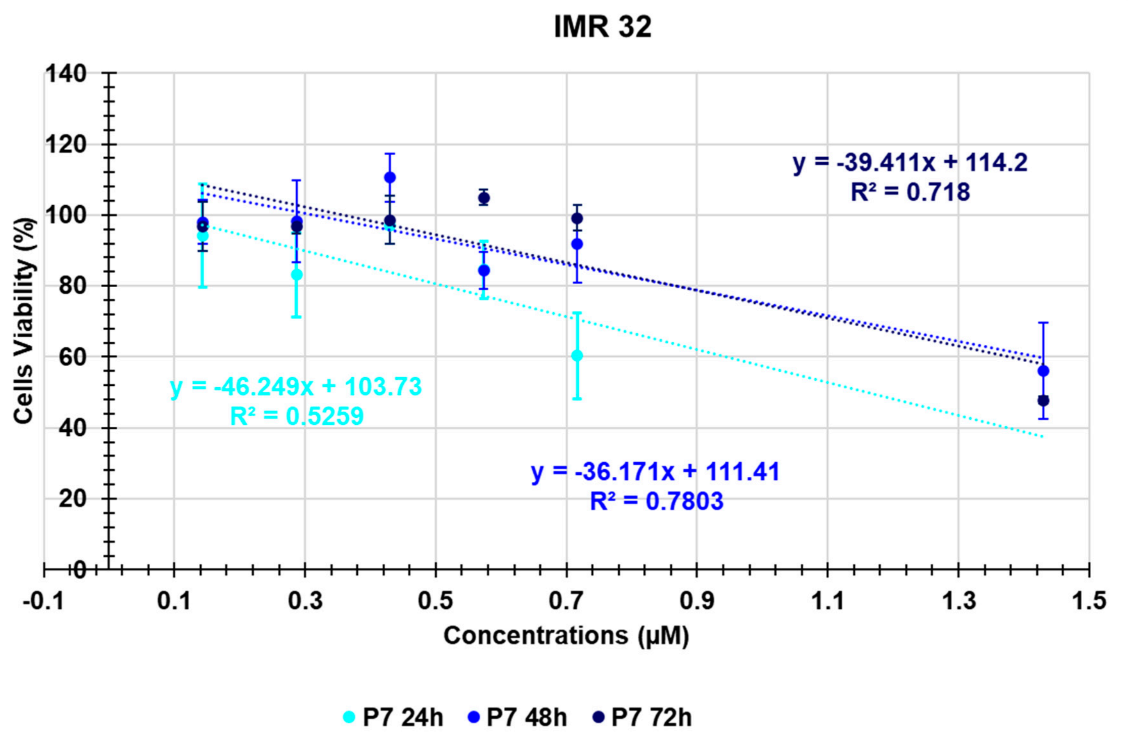

Figure S15. Dose-dependent cytotoxicity activity of P7 NPs at 24 h (light blue), 48 h (blue), and 72 h (dark blue) towards IMR-32 cells in the range 0.14-1.44 μM.

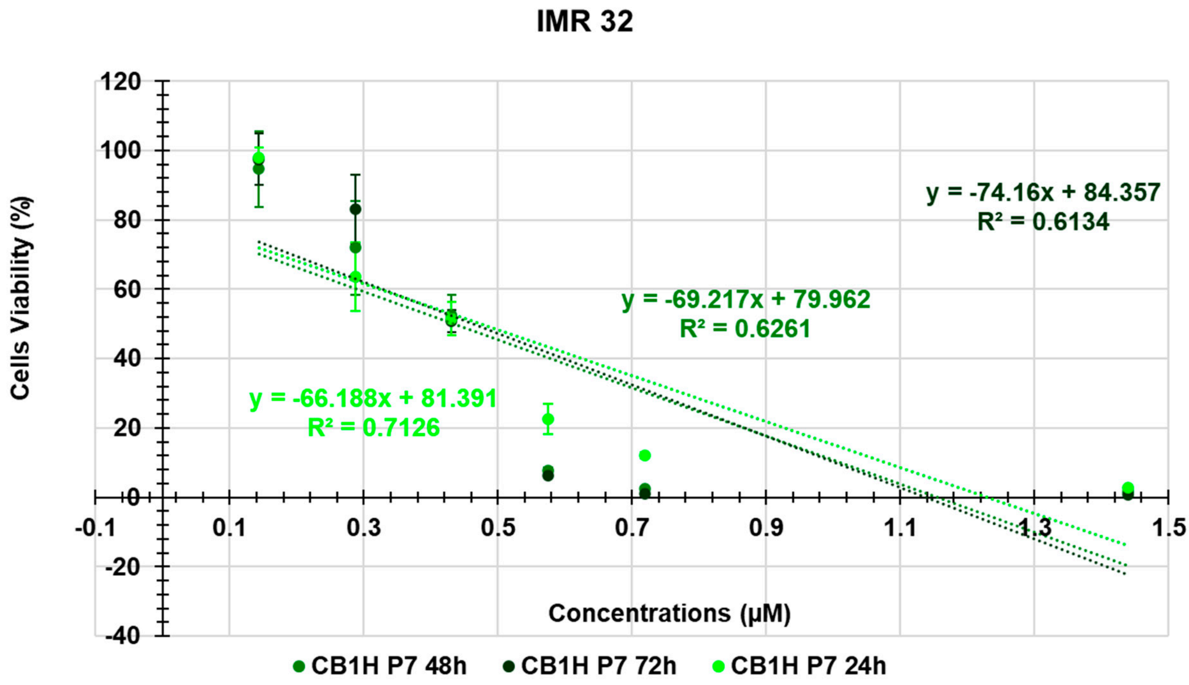

Figure S16. Dose-dependent cytotoxicity activity of CB1H-P7 NPs at 24 h (light green), 48 h (green), and 72 h (dark green) towards IMR-32 cells in the range 0.14-1.44 μM.

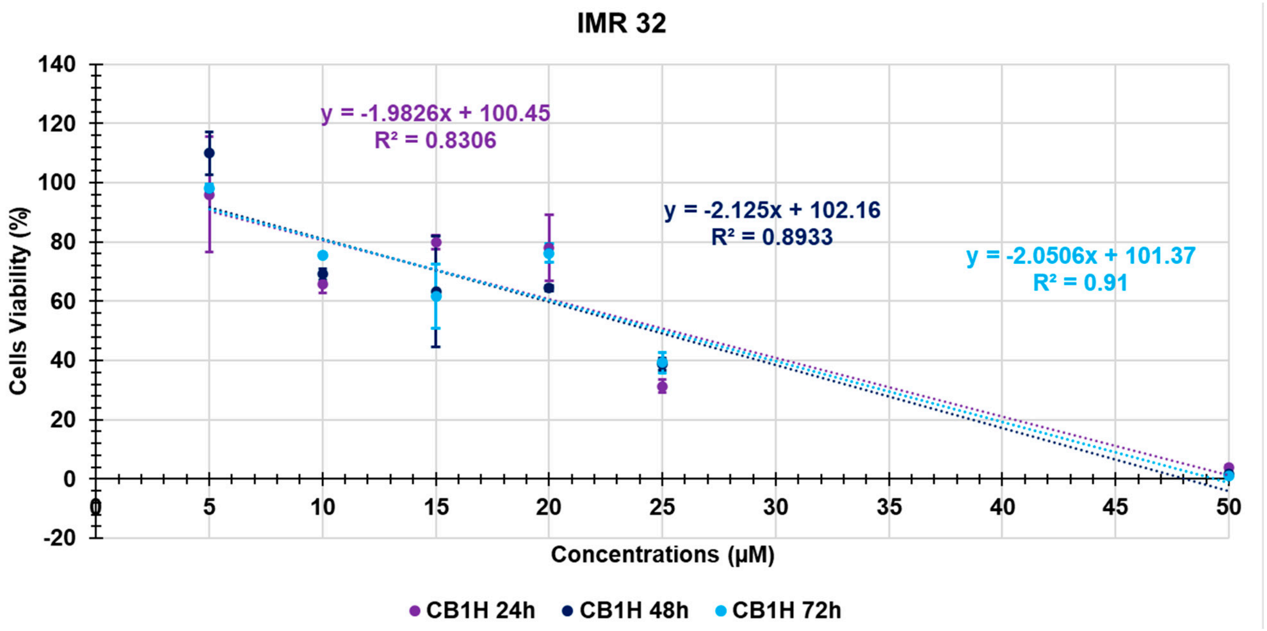

Figure S17. Dose-dependent cytotoxicity activity of CB1H at 24 h (purple), 48 h (blue), and 72 h (light blue) towards IMR-32 cells in the range 5-50 μM.

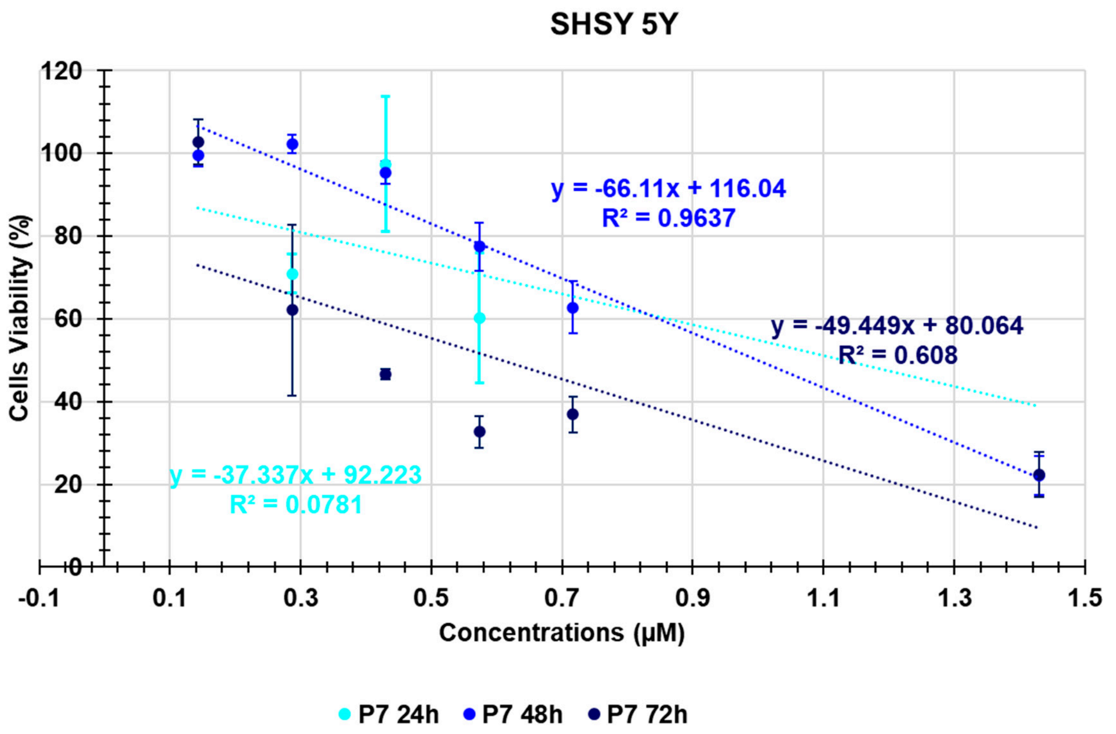

Figure S18. Dose-dependent cytotoxicity activity of P7 NPs at 24 h (light blue), 48 h (blue), and 72 h (dark blue) towards SHSY 5Y cells in the range 0.14-1.44 μM.

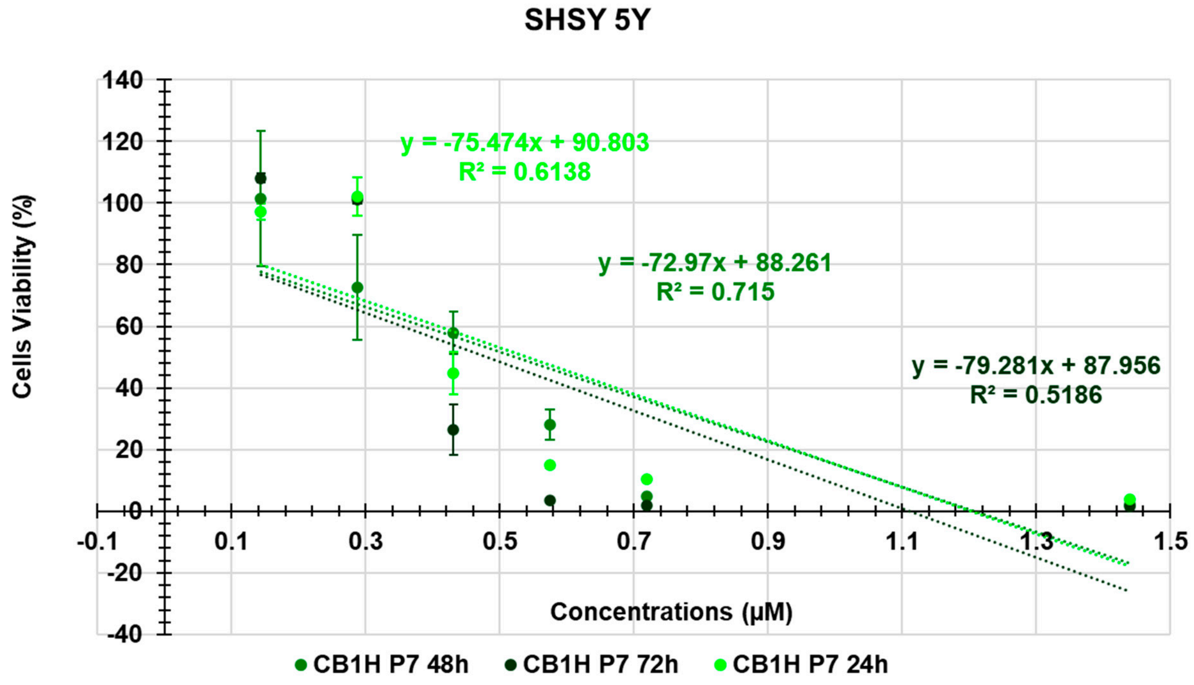

Figure S19. Dose-dependent cytotoxicity activity of CB1H-P7 NPs at 24 h (light green), 48 h (green), and 72 h (dark green) towards SHSY 5Y cells in the range 0.14-1.44 μM.

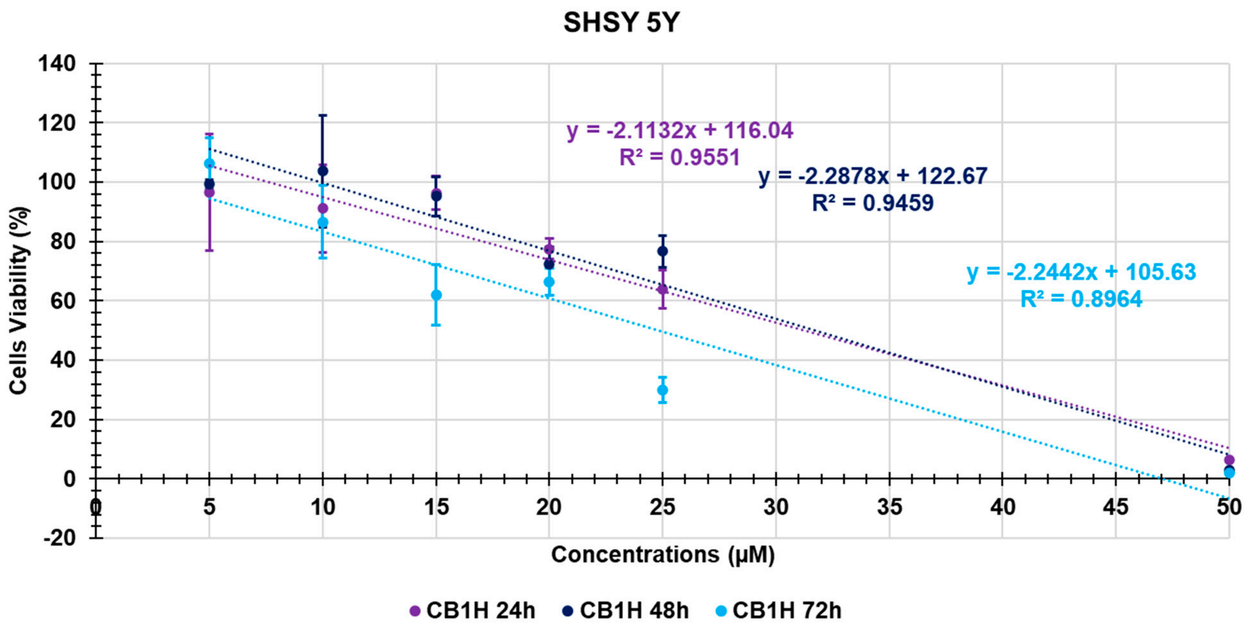

Figure S20. Dose-dependent cytotoxicity activity of CB1H at 24 h (purple), 48 h (blue), and 72 h (light blue) towards SHSY 5Y cells in the range 5-50 μM.

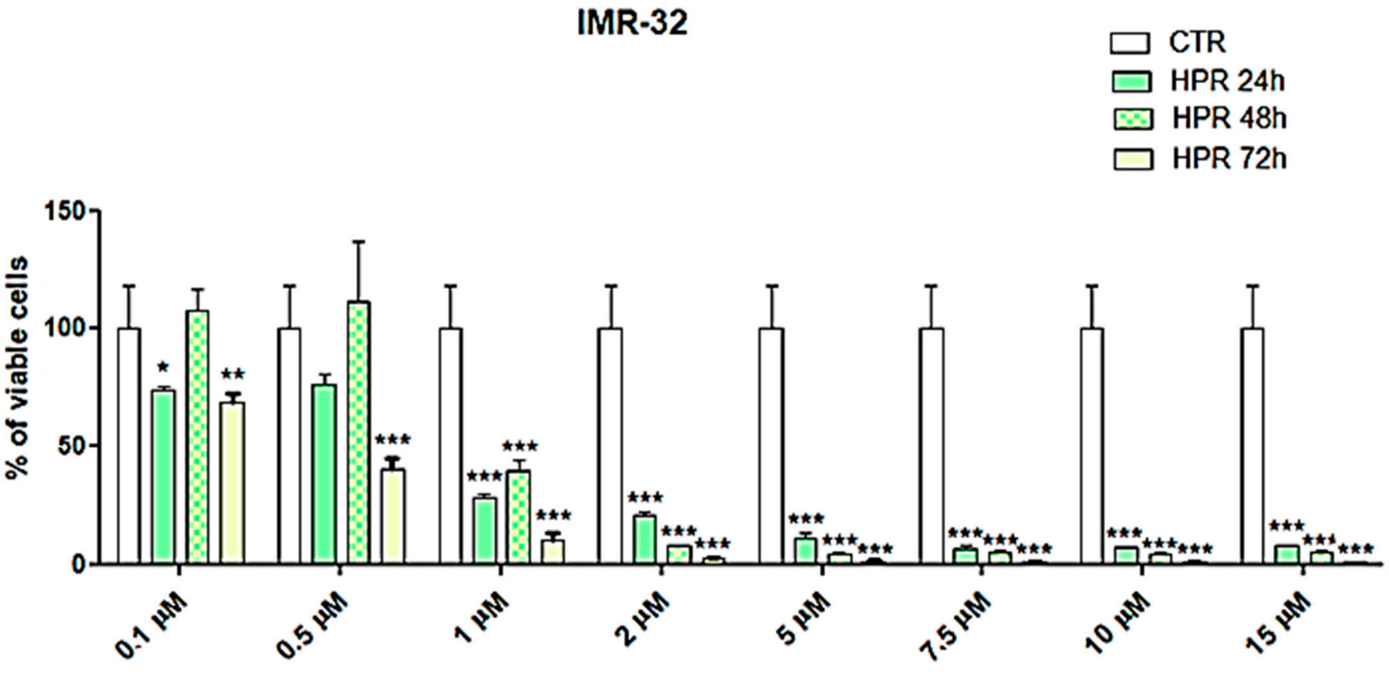

Figure S21. Dose- and time-dependent cytotoxicity activity of 4-HPR at 24 h, 48 h, and 72 h towards IMR-32 cells. Significance refers to control (p > 0.05 ns; p < 0.05 *; p < 0.01 **; p < 0.001 ***).

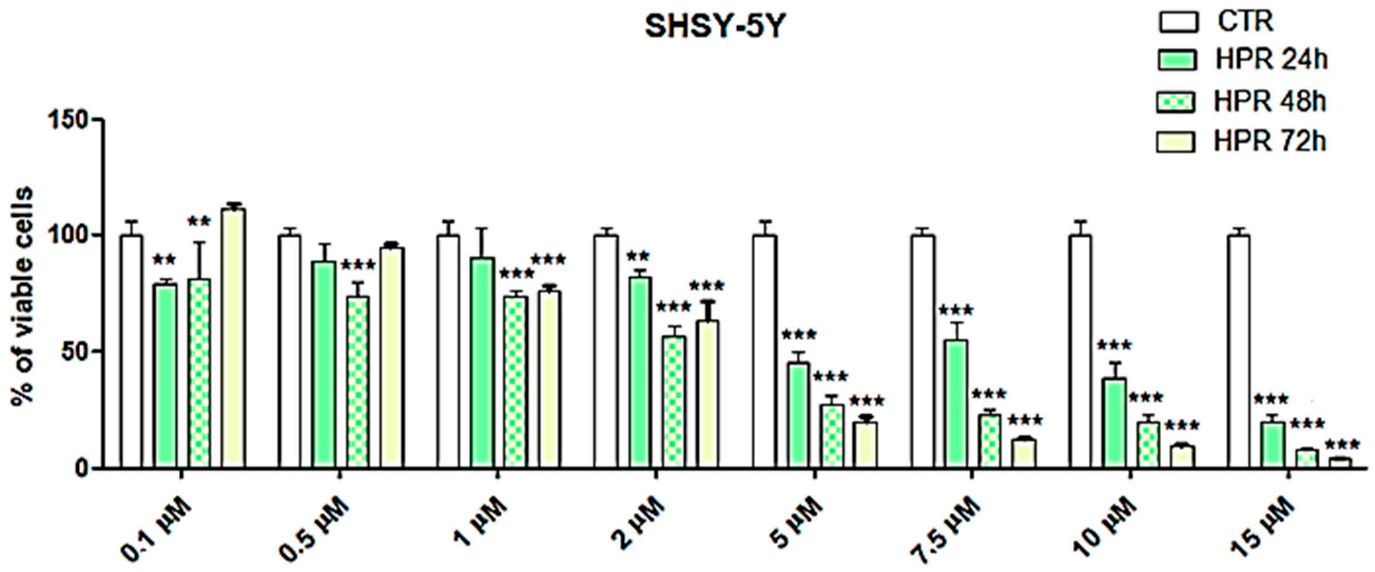

Figure S22. Dose- and time-dependent cytotoxicity activity of 4-HPR at 24 h, 48 h, and 72 h towards SHSY-5Y cells. Significance refers to control (p > 0.05 ns; p < 0.05 *; p < 0.01 **; p < 0.001 ***).

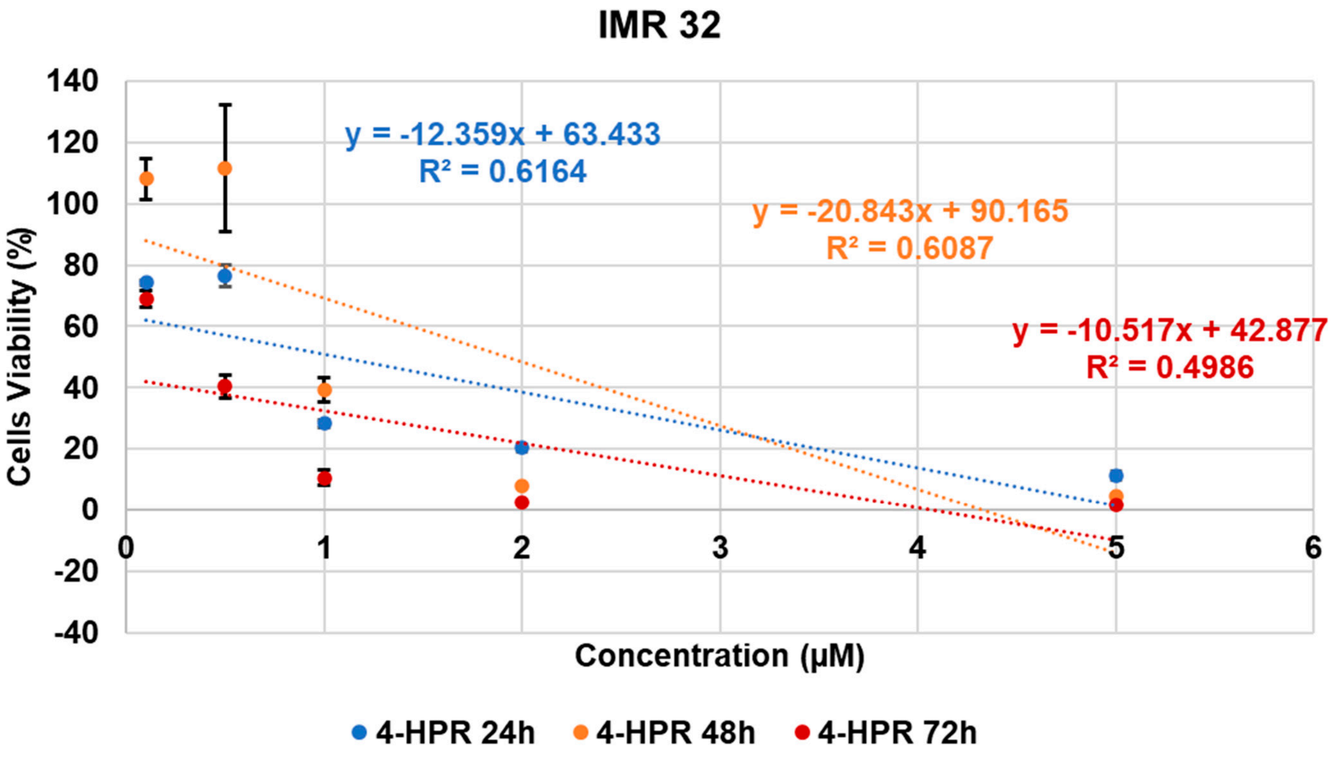

Figure S23. Dose-dependent cytotoxicity activity of 4-HPR at 24 h (blue), 48 h (orange), and 72 h (red) towards IMR-32 cells in the range 0.11-5 µM. Concentrations over 5 µM were not considered because cells viability did not significantly change further.

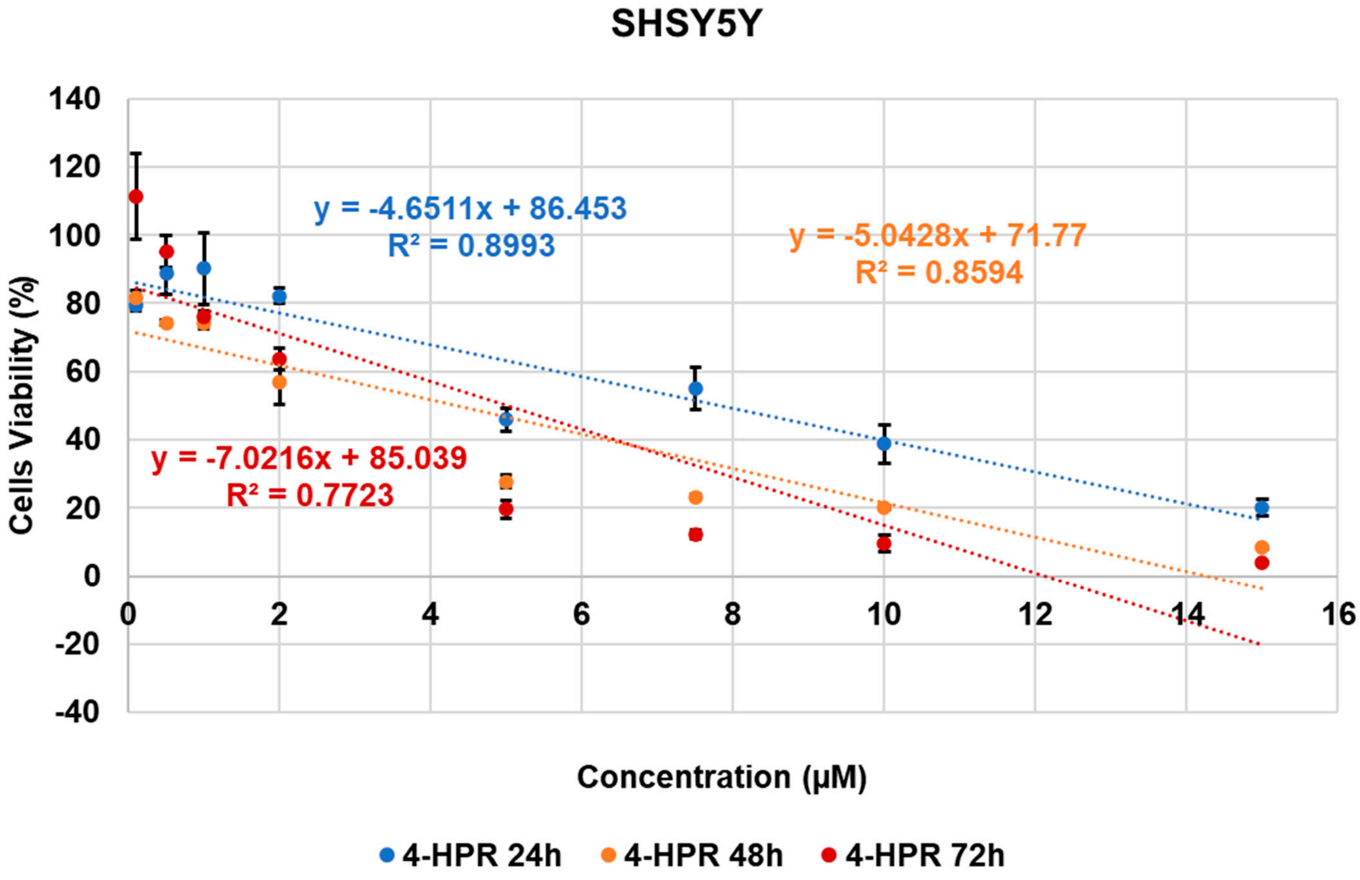

Figure S24. Dose-dependent cytotoxicity activity of 4-HPR at 24 h (blue), 48 h (orange), and 72 h (red) towards SHSY 5Y cells in the range 0.1-15 µM.

Section S3. Physicochemical Characteristics of Pyrazole-loaded Nanoparticles.

Table S4. Main characteristic of BBB4-G4K NPs [16].

Analysis	BBB4-G4K NPs	
FTIR<br>[cm^{-1}]	Bands Attribution<br>3500–3000 (NH_3^+ dendrimer, OH stretching BBB4)<br>2985, 2880 (alkyl groups of dendrimer and BBB4)<br>1736 (C=O stretching esters of dendrimer)<br>1220, 1051 (C-O stretching esters of dendrimer)<br>697 (C-Br stretching of BBB4)	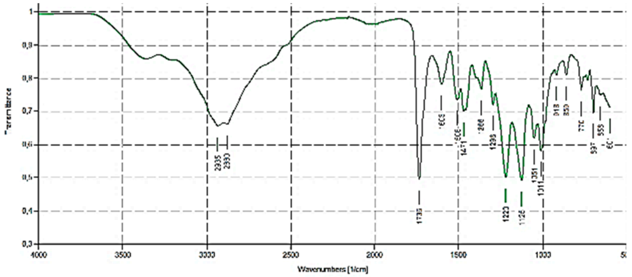
^{1}H NMR<br>(400 MHz, CD_3OD)<br>[ppm]	Signals attribution<br>**1** = 423 H (CH_3 of G4K + $CH_2CH_2CH_2$ of lys)<br>**2** = 96 H ($CH_2NH_3^+$ of lys)<br>**3** = 120H (CH_2 of BBB4 + $CHNH_3^+$ of lys + CH_2 of BBB4)<br>**4** = 186 H (CH_2O of G4K)<br>**5** = 144 H (CH= of phenyl rings)<br>**6** = 36 H (CH= of phenyl rings)	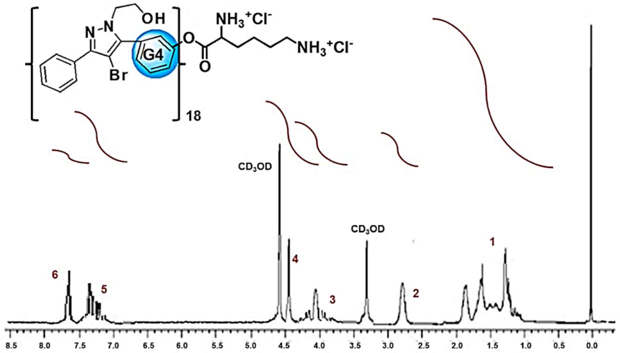
HPLC	DL (%)	28.8 ± 1.2

	EE (%)	39.0 ± 1.6
^{1}H NMR	MW	21,175.8
DL% by HPLC		21,072.6 ± 240.2
Scanning Electron Microscopy (SEM)	Morphology<br>Average Size	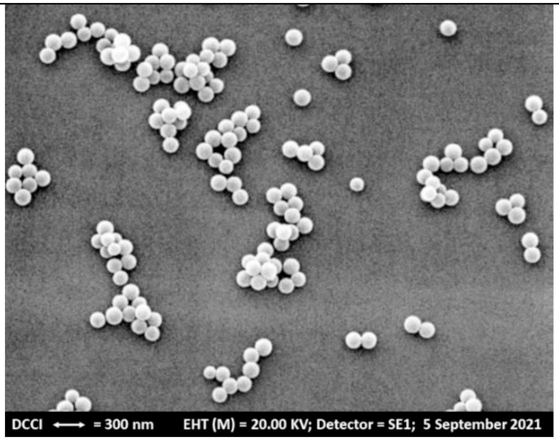
DLS [1] Analysis	Z-Ave [2,5] (nm)<br>PDI [3,5]	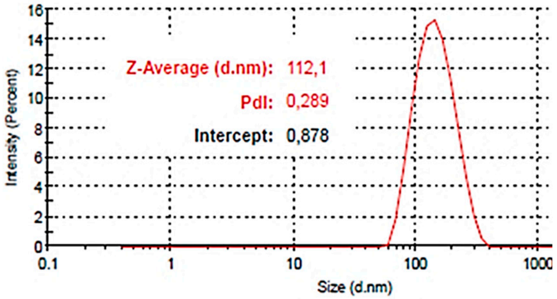

	ζ-p [4,5](mV)	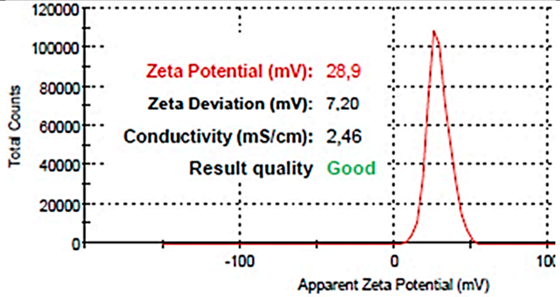
Solubilization Essay [§]	Water-Solubility (mg/mL)	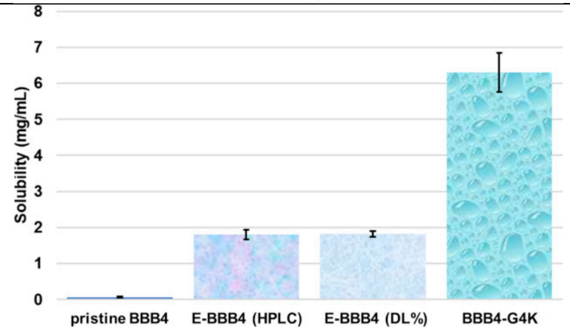
Dialysis Method (HPLC)	Cumulative Release (%, 24h)	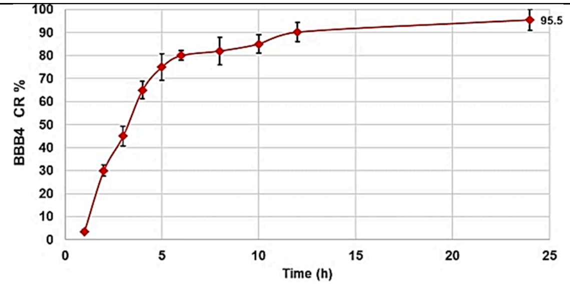

	Mathematical Model	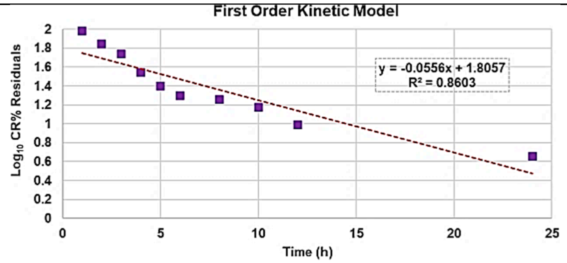
	Mechanism	Drug release rate is concentration-dependent
G4K Cytotoxicity<br>(HeLa Cells)	Cell Viability (%)<br>(0–100 μM)	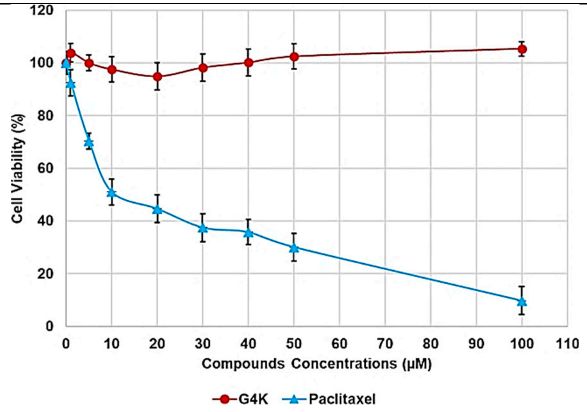

Potentiometric Titration [#]	Protonation Profile	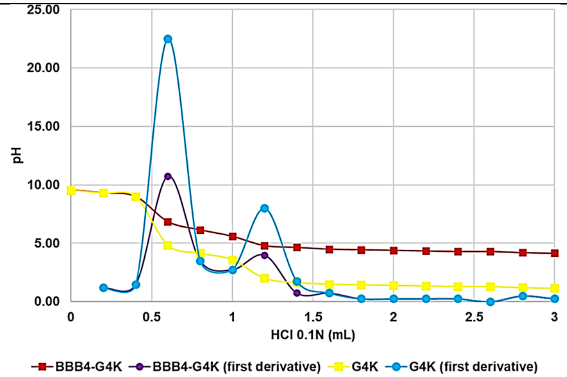

[1] dynamic light scattering; [2] hydrodynamic diameters of particles; [3] polydispersity indices; [4] measure of the electrical charge of particles suspended in the liquid of acquisition (water); [5] correspondent values for G4K = 333.4 ± 24.6, 0.286 ± 0.040, +66.1 ± 4.7; [§] the image also shows the water solubility of untreated BBB4, nanoengineered BBB4 released in water, and BBB4 contained in NPs; Paclitaxel = positive control; [#] the image also shows the titration curve and the first derivative of G4K.

Table S5. Main physicochemical properties of CB1H-P7 NPs.

<table>
<tr><th>Analysis</th><th colspan="2">CB1H-P7 NPs</th></tr>
<tr><td>FTIR [cm $^{-1}$]</td><td colspan="2">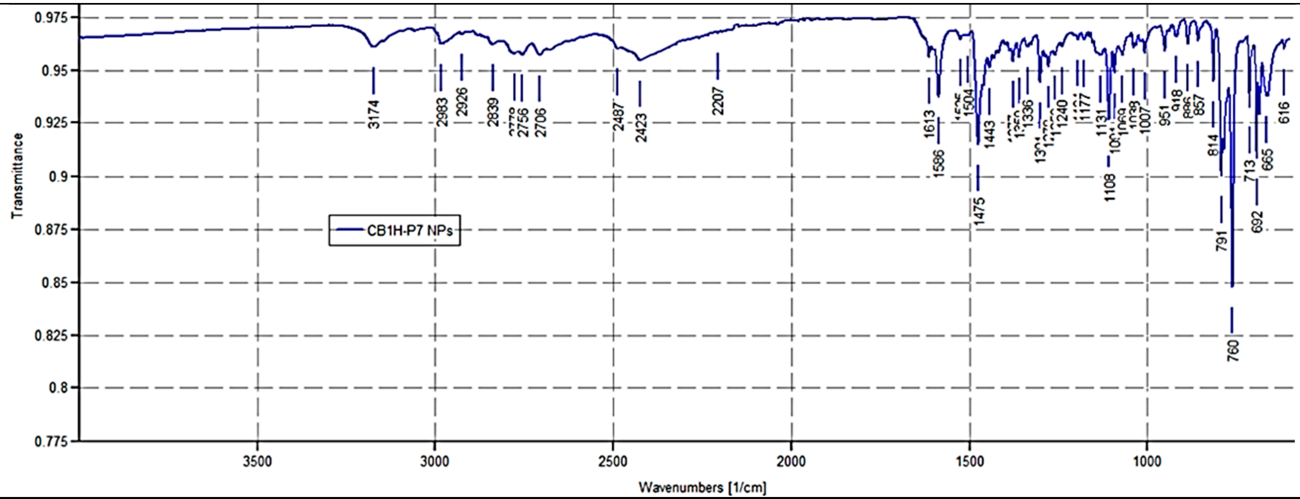
</td></tr>
<tr><td>Principal Components Analysis (PCA)</td><td colspan="2">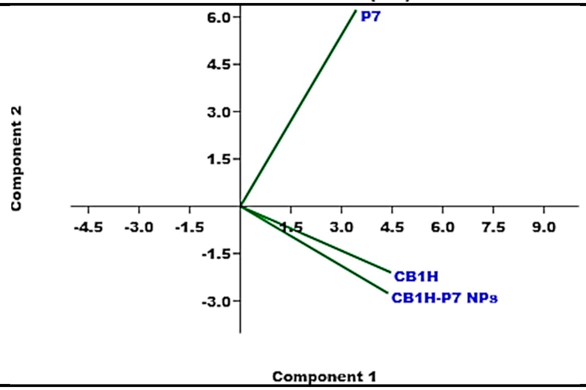
</td></tr>
<tr><td>UV-Vis</td><td>Ultraviolet Spectrum</td><td>λ_{abs} = 250 nm</td></tr>
<tr><td>UV-Vis</td><td>DL (%)</td><td>48.6±1.4</td></tr>
</table>

	EE (%)	81.5±2.50
DL% (UV-Vis)	MW	26,623.9±260.3
Scanning Electron Microscopy (SEM)	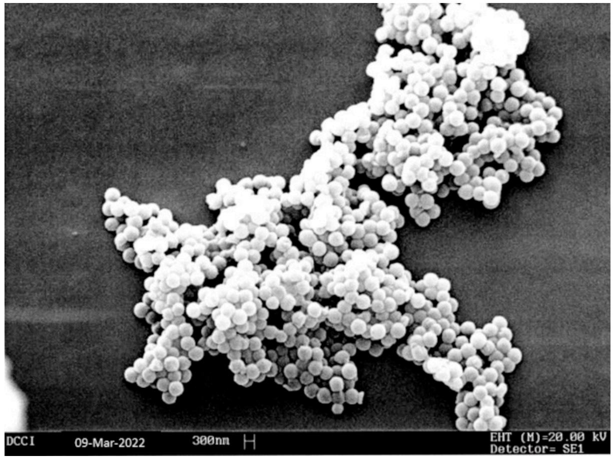 P7 (342.9±57.2 nm)	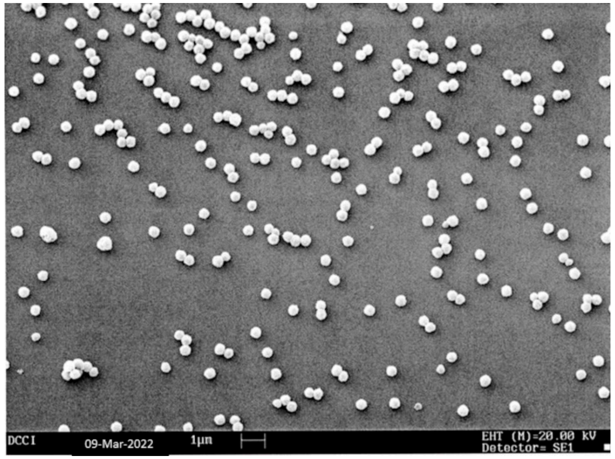 CB1H-P7 NPs (332.8±67.9 nm)
DLS [1] Analysis	Z-Ave [2] (nm)<br>PDI [3]<br>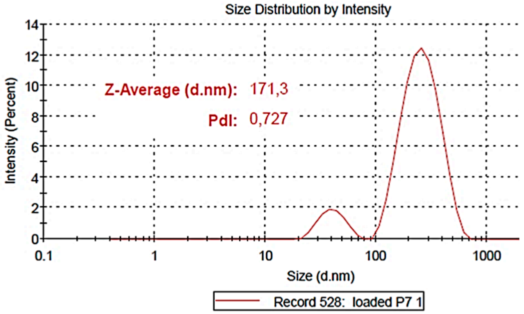	142.9±20.1<br>0.626±0.071

	ζ-p [4] (mV)	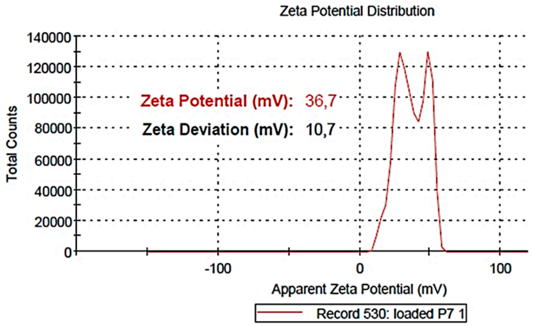	+36.7±10.7
Dialysis Method (UV-Vis)	Cumulative Release (%, 24h)		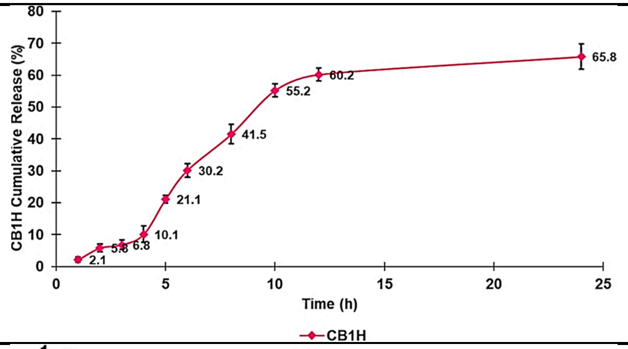
	Mathematical Model		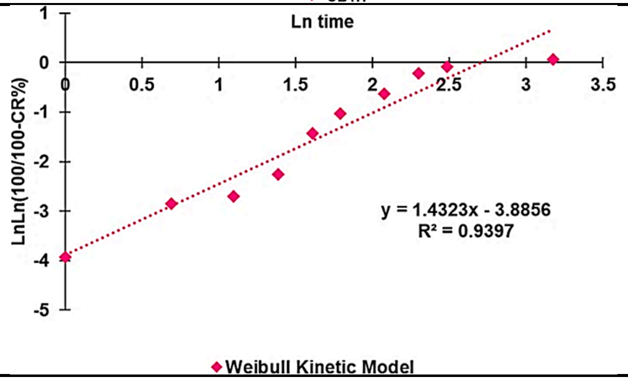
	Mechanism		(β>1) Complex Mechanisms

Potentiometric Titration[#]	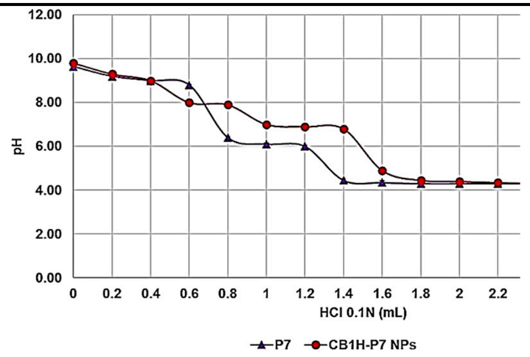 Potentiometric Tritration	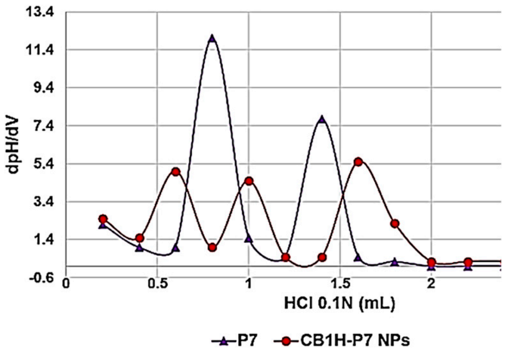 First Derivative

[1] dynamic light scattering; [2] hydrodynamic diameters of particles; [3] polydispersity indices; [4] measures of the electrical charge of particles suspended in the liquid of acquisition (water).

Section S4. Conclusions.

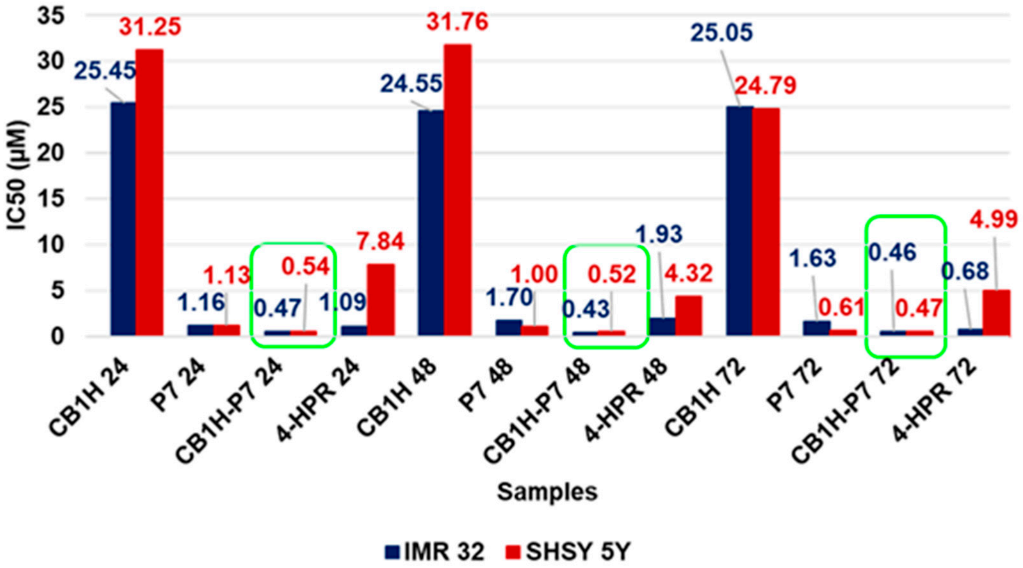

Figure S25. Comparison between the IC_{50} values of CB1H, P7, CB1H-P7 and 4-HPR at 24 h, 48 h, and 72 h towards IMR-32 and SHSY 5Y cells.

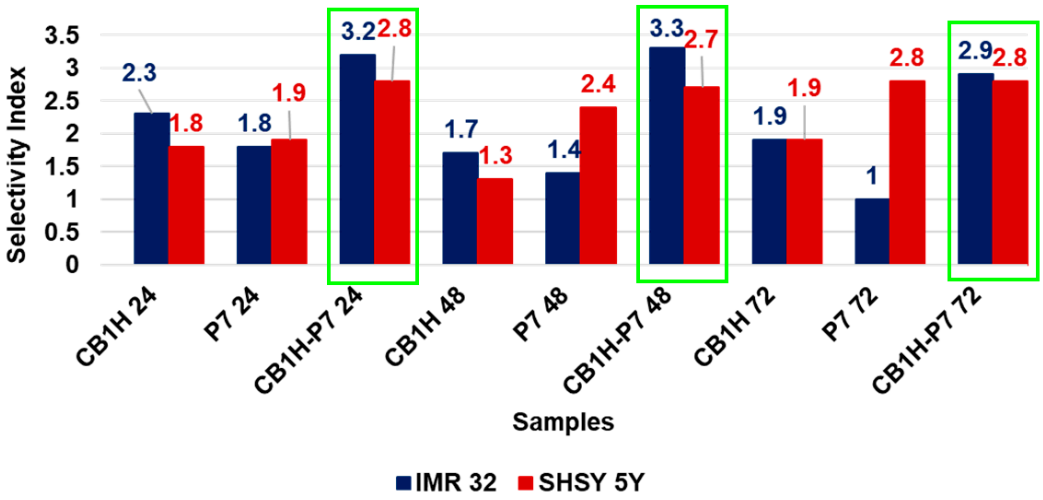

Figure S26. Comparison between the SI values of CB1H, P7, and CB1H-P7 at 24, 48 and 72 h towards IMR-32 and SHSY 5Y cells.
